# Ecological Biogeography of the Terrestrial Nematodes of Victoria Land, Antarctica

**DOI:** 10.3897/zookeys.419.7180

**Published:** 2014-06-23

**Authors:** Byron J. Adams, Diana H. Wall, Ross A. Virginia, Emma Broos, Matthew A. Knox

**Affiliations:** 1 Department of Biology, and Evolutionary Ecology Laboratories, Brigham Young University, Provo, UT 84602; 2 Department of Biology and Natural Resource Ecology Laboratory, Colorado State University, Fort Collins, CO 80523-1499; 3 Environmental Studies Program, Dartmouth College, Hanover, NH 03755; 4 Natural Resource Ecology Laboratory, Colorado State University, Fort Collins, CO 80523-1499

**Keywords:** Biodiversity, dispersal, climate change, *Eudorylaimus*, freeliving nematodes, *Geomonhystera*, habitat suitability, invasive species, *Panagrolaimus*, *Plectus*, *Scottnema*, soil

## Abstract

The terrestrial ecosystems of Victoria Land, Antarctica are characteristically simple in terms of biological diversity and ecological functioning. Nematodes are the most commonly encountered and abundant metazoans of Victoria Land soils, yet little is known of their diversity and distribution. Herein we present a summary of the geographic distribution, habitats and ecology of the terrestrial nematodes of Victoria Land from published and unpublished sources. All Victoria Land nematodes are endemic to Antarctica, and many are common and widely distributed at landscape scales. However, at smaller spatial scales, populations can have patchy distributions, with the presence or absence of each species strongly influenced by specific habitat requirements. As the frequency of nematode introductions to Antarctica increases, and soil habitats are altered in response to climate change, our current understanding of the environmental parameters associated with the biogeography of Antarctic nematofauna will be crucial to monitoring and possibly mitigating changes to these unique soil ecosystems.

## Introduction

Understanding the global distribution of biodiversity is critical for studying the evolution, ecology and dynamics of ecosystems and to address how global scale changes in climate, invasive species, and land use will affect ecosystems, ecosystem services, and subsequently, people. Antarctic terrestrial ecosystems might seem less sensitive to global change because this polar desert has low species diversity distributed across a limited area of biologically active ice-free land, comprising less than 0.32% of the continent’s 14 million km^2^ (Chown and Convey 2007). However, terrestrial ecosystems of Antarctica are not immune to global changes (Adams et al. 2009; Chown et al. 2012b). Small changes in polar climate are amplified through biophysical feedbacks leading to biologically significant alterations in soil habitats and their communities (Doran et al. 2002; Nielsen et al. 2011a). The low species diversity of Antarctic soils makes them uniquely suited for studying the relationships between soil biodiversity and ecosystem functioning, and identifying how global changes may affect species level changes in biodiversity, community composition and distribution (Barrett et al. 2008; Simmons et al. 2009). Measures to conserve, manage and sustain ecosystem functioning in Antarctic and Earth’s other low diversity terrestrial environments will rely on knowledge of species diversity, distributions, and their role in ecosystem processes (Adams et al. 2006; Barrett et al. 2008; Wall 2004).

Aboveground, the diversity and biogeography of terrestrial flora (mosses, lichens and liverworts) has been recently assessed and used to further refine the geographic floral regions of Antarctica (Peat et al. 2007). It is well known that the warmer maritime and subantarctic ecosystems have higher precipitation, organic soils, a more diverse and abundant vegetation (Bölter et al. 2002; Maslen 1979; Nielsen et al. 2011b; Peat et al. 2007) and greater soil faunal diversity (including earthworms and beetles) than continental Antarctica (Block and Christensen 1985; Chown and Van Drimmelen 1992). For example, the northern maritime Antarctic has 100-115 moss and *c.* 350 lichen species compared to continental Antarctica’s 20–30 moss and *c.* 90 lichen species (Peat et al. 2007). Throughout Victoria Land vascular plants are absent and fauna are reduced to only a few soil groups and are represented by a patchy spatial distribution of protozoans, nematodes, rotifers, tardigrades, springtails (Collembola), and mites (Acarina) (Adams et al. 2006; Bamforth et al. 2005; Frati et al. 1997; Moorhead et al. 1999; Stevens and Hogg 2002; Virginia and Wall 1999).

Nematoda are a major component of soil food webs in all terrestrial ecosystems including the exposed lands of Antarctica, though their spatial distribution and abundance are highly heterogeneous. In more productive ecosystems, they typically have much higher diversity (Wall Freckman and Virginia 1998) than the Antarctic (Boag and Yeates 1998; Bunt 1954; Maslen 1981). For example, 431 nematode species were recorded from a Cameroon tropical forest ecosystem, with a maximum of 89 species found in 200 individuals enumerated in a soil core (Bloemers et al. 1997). In contrast, the diversity of nematodes in all of Antarctica, including the continental, maritime, and Sub- Antarctic is 54 nematode species, of which only *c.* 22 species, all endemic, occur on the ice-free terrestrial areas of the continent (Andrássy 1998; Andrássy 2008).

In Antarctica, soil nematodes have been studied primarily in localized and easily accessible areas largely centered around research bases and concentrated on the Antarctic peninsula and islands of the maritime Antarctic and further south in ice-free areas. As a consequence there is relatively little known of their regional biogeography or of the habitats that are suitable for functioning communities. Additionally, there are many remote inland ice-free areas which have yet to be sampled (Convey 1996; Wall 2005), adding to questions on how widespread species are, and whether species rich communities and habitats exist in the more extreme climate zones of the continent.

**Figure 1.**
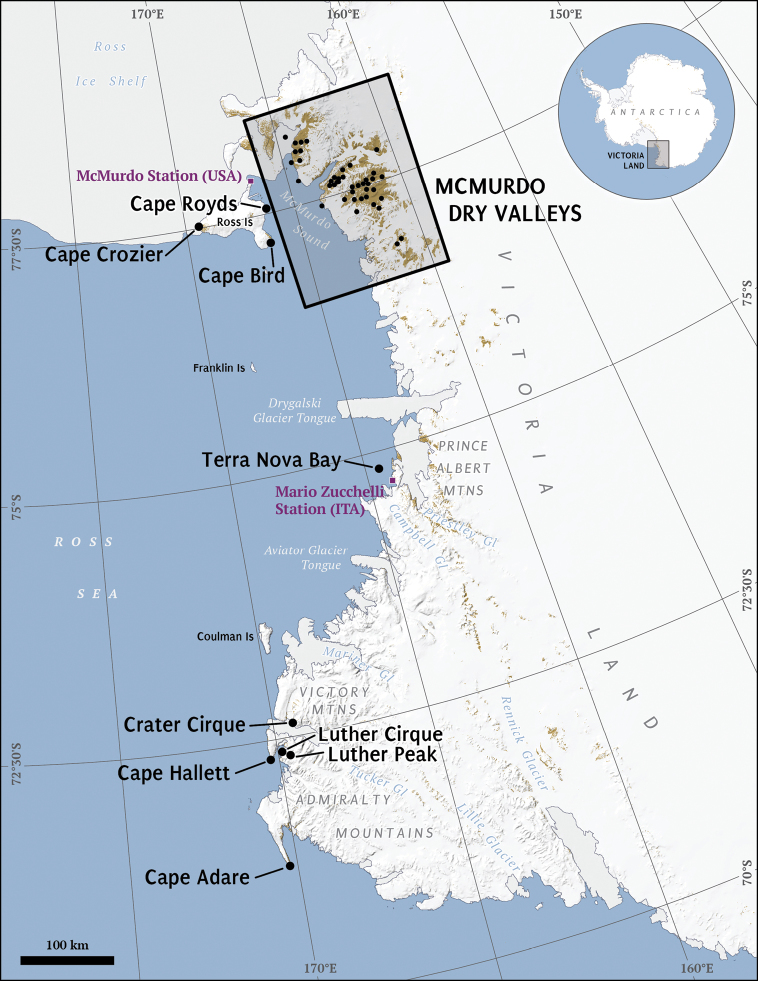
Victoria Land, Antarctica. Labeled areas represent study locations and major geographic features referenced in the tables and text. Box inset of the McMurdo Dry Valleys is rotated 180 °and expanded in Figure 2.

**Figure 2.**
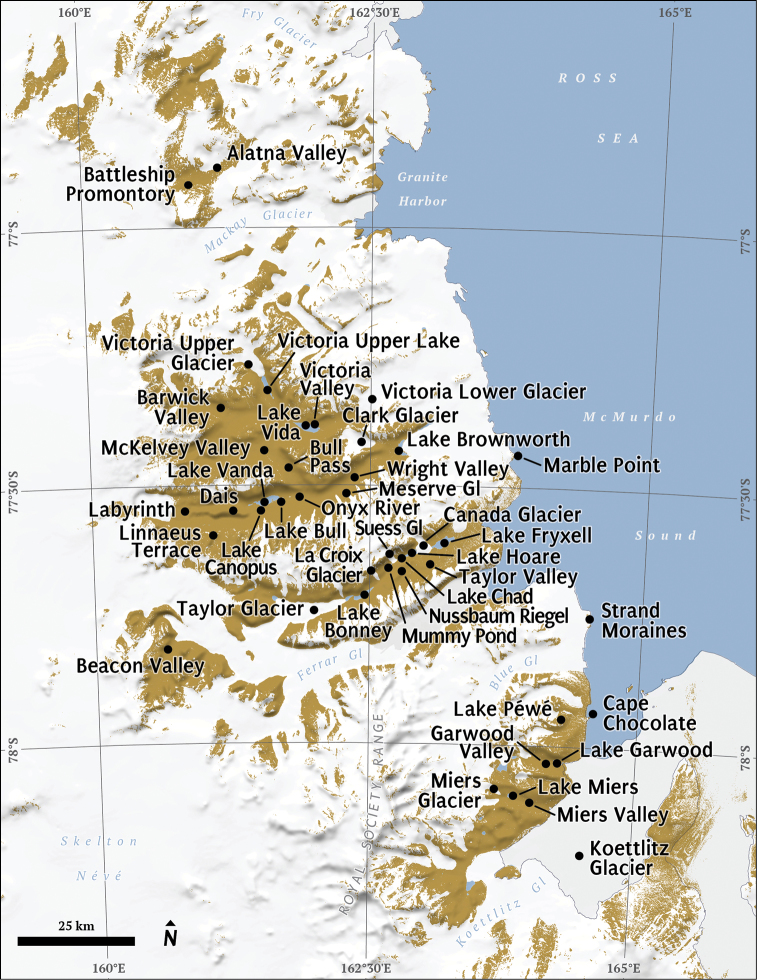
McMurdo Dry Valleys, Antarctica. Labeled areas represent study locations and major geographic features referenced in the tables and text.

Regional to continental-scale descriptions of the Antarctic nematofauna have pointed to a paucity of distributional records for much of the continent (Andrássy 1998; Velasco-Castrillón and Stevens 2014). Amongst all regions of Antarctica, Victoria Land is arguably the most intensively studied (Adams et al. 2006). Victoria Land is “ that part of Antarctica which fronts on the western side of the Ross Sea, extending southward from about 70°30'S to 78°00'S, and westward from the Ross Sea to the edge of the polar plateau” (USGS 2003). Here, we synthesize information on the nematode biodiversity, geographic distribution and soil and sediment habitats of the terrestrial nematodes in Victoria Land, Antarctica. Much of this information comes from a series of studies to assess nematode diversity and distribution begun in austral summer 1989–1990 by Wall (formerly Freckman) and Virginia and extending to the present as part of the McMurdo Dry Valley Long Term Ecological Research program funded by the US National Science Foundation (www.mcmlter.org). We report on findings of these studies through 2004 which captures most of the biodiversity information gathered by this research group, whereas more recent research has focused on nematode species response to climate change and soil resource manipulations (Ayres et al. 2010; Doran et al. 2002; Simmons et al. 2009). For purposes of our synthesis, we define two areas, Northern Victoria Land - the area from about 70°30'S to about 76°S, encompassing Terra Nova Bay, Edmonson Point and Cape Hallett (Figure 1); and Southern Victoria Land - the area from about 76°S to about 78°S including all of the McMurdo Dry Valleys and nearby coastal regions (Adams et al. 2006) (Figure 2).

The McMurdo Dry Valleys (76°5'to 78°5'S, 160°0' to 164°0'E) are located along the TransAntarctic Mountains in Southern Victoria Land and comprise about 4,800 km^2^ of ice-free land and have different geo/ecological legacies and climatic conditions (Lyons et al. 2000; Moorhead et al. 1999). They are the oldest, driest and coldest deserts on earth (Beyer et al. 1999; Campbell et al. 1998; Fountain et al. 1999). Annual precipitation is less than 10 cm water equivalent, most of which sublimates before it melts (Doran et al. 2002; Fountain et al. 1999). Mean annual air temperature is –20°C (Fountain et al. 1999) and surface soil temperature ranges from -59 °C in winter to 26 °C for short periods during summer (Doran et al. 2002). No vertebrate animals or vascular plants are present and mosses and lichens are rare and mostly confined to ephemeral meltponds, streams and lake moats (Cameron et al. 1970; Horowitz et al. 1972; Kappen 1993). Across the region soils are poorly developed, coarse textured (95 to 99% sand by weight) (Bockheim 1997), low in organic carbon (<1%) (Burkins et al. 2000), saline, and have low biological activity compared to warmer ecosystems (Ball et al. 2009; Barrett et al. 2006a; Parsons et al. 2004). Nematodes are the dominant soil invertebrate, but many soils (~35%) lack extractable soil invertebrates and approximately 50% of McMurdo Dry Valleys soils that contain invertebrates have only one invertebrate species (Freckman and Virginia 1997; Wall Freckman and Virginia 1998).

The distributions of the Dry Valley metazoan species are associated with specific sites and correlate to soil habitat differences in organic matter content, moisture and salinity, and microclimate differences encountered over environmental gradients of coastal to interior sites, latitude, and soil chronosequences and differences in glacial tills (Barrett et al. 2006a).

Coastal areas of Victoria Land are a moister environment than the Dry Valleys and are habitat for birds and marine mammals (e.g. skua gulls, penguins, and seals). Penguin rookeries are associated with ornithogenic soils with significant inputs of carbon and nitrogen transferred from the marine environment to the soil (Bargagli et al. 1997). Ornithogenic soils are the only soils south of the Antarctic Circle containing high concentrations (14–21%) of organic matter (Campbell and Claridge 1966; Heine and Speir 1989). However, even with high C and N availability these soils often have lower nematode diversity than soils of the Dry Valleys, probably owing to very high concentrations of salts and soil compaction and cementing (Porazinska et al. 2002a; Sinclair 2001).

Each of the unique soil ecosystems of Victoria Land imposes considerable physiological constraints on nematode life history traits, requiring adaptive responses to freeze/thaw cycling, osmotic and desiccation stress, and a short growing season (Convey 1996). Nematode responses include cryoprotective dehydration via anhydrobiosis (Adhikari et al. 2009; Adhikari and Adams 2011; Crowe et al. 1992), as well as tolerance to inter and intracellular freezing (Adhikari et al. 2010; Wharton 2003, 2010) and multiyear lifecycles (de Tomasel et al. 2013; Overhoff et al. 1993; Yeates et al. 2009). In addition to stress survival, anhydrobiosis also facilitates long-distance aeolian dispersal (Barrett et al. 2006a), an important mechanism implicated in explanations of their geographic distributions and population genetic structure (Adams et al. 2006; Courtright et al. 2000). All of the nematodes of Victoria Land are inferred to be microbivores with the exception of *Eudorylaimus*, which is omnivorous (Yeates et al. 1993) (but see Wall 2007).

Nematodes were first collected in Victoria Land by the British ‘Discovery’ expedition of 1901-1903, from Discovery Bay, South Victoria Land and described by Steiner (1916) as *Dorylaimus antarcticus* (syn. *Eudorylaimus antarcticus* (Yeates 1970)). The nematodes of Victoria Land then remained largely unstudied for over half a century, until the work of Yeates (1970) and Timm (1971). Between them, these two papers described or redescribed all Victoria Land genera of the time and laid the foundation for future taxonomic work. Yeates (1970) recorded *Plectus* from southern coastal Victoria Land and synonymized *Dorylaimus antarcticus* and *Antholaimus antarcticus* with *Eudorylaimus antarcticus*. However, subsequent studies have described further *Eudorylaimus* species from continental Antarctica: *Eudorylaimus glacialis* (Andrássy 1998), *Eudorylaimus nudicaudatus* (Heyns 1993) and *Eudorylaimus shirasei* (Kito et al. 1996), *Eudorylaimus quintus* (Andrássy 2008) and *Eudorylaimus sextus* (Andrássy 2008). Due to the taxonomic uncertainty of early accounts (Adams et al. 2006), we will henceforth use *Eudorylaimus* sp. in reference to all previous reports of distribution. Timm (1971) synonymized *Plectus murrayi* with *Plectus antarcticus* (de Man 1904) and studied parts of southern and northern coastal Victoria Land and the McMurdo Dry Valleys. He also re-described three known species: *Eudorylaimus antarcticus* (Steiner 1916), *Monhystera villosa* (Bütschli 1873) and *Plectus frigophilus* (Kirjanova 1958), and described two new species, *Scottnema lindsayae* and *Panagrolaimus davidi*. *Monhystera villosa* was later synonymized with *Geomonhystera antarcticola* (Andrássy 1998). These early studies focused exclusively on the identification and description of nematode species and not their ecologies.

In the McMurdo Dry Valley Region, most nematological studies have investigated the diversity, ecology and distribution patterns of up to three nematode genera; *Eudorylaimus*, *Plectus*, *Scottnema* (Adams et al. 2006), while the coastal areas of Victoria Land remain less well known (Adams et al. 2006; Bargagli et al. 1997; Barrett et al. 2006a; Porazinska et al. 2002a; Raymond et al. 2013a; Sinclair and Sjursen 2001; this paper; Timm 1971; Vinciguerra 1994). Our effort here is a synthesis of the biogeographic distribution of nematodes in Victoria Land and a consideration of the soil habitats that are associated with nematode distribution, diversity and abundance.

## Materials and methods

Based on published and unpublished data, we summarized biogeographic information on the species represented within each nematode genus described in Victoria Land. In addition to published papers, we present information obtained from data on soil, and lake and stream sediment samples collected throughout Victoria Land, by the authors and team members during the austral summers between and including 1990 and 2004. Data referred to as “this study (year)” were derived from nematode soil extraction procedures optimized for Antarctic soils and all nematodes were identified to species (Freckman and Virginia 1993). Frozen soils from these samples are archived at the Wall lab in the Department of Biology at Colorado State University, Fort Collins, CO, USA. Formalin-preserved extracted specimens from these soils are archived in the meiofauna collection of the Monte L. Bean Life Science Museum at Brigham Young University, Provo, UT, USA. Non-occurrences are not reported but can be extrapolated from Tables 1–5. A brief summary of published information on the ecology of each genus is also provided (Table 6).

**Table 1.** Biogeographic distribution of *Scottnema lindsayae* in Victoria Land, Antarctica. NP = not published. NA = specific coordinates not available within the named locale identified above. For references to “this paper”, the year collected refers to the year at the beginning of the austral summer in which samples were collected at 0-10 cm depth unless otherwise indicated. For abundance, ‘Present’ indicates no abundance information available, Low = >0 to 20 nematodes per kg dry soil, M-low = 21 to 200 nematodes per kg dry soil, Medium = 201 to 600 nematodes per kg dry soil, M-high = 601 to 1000 nematodes per kg dry soil, High = 1001 to 2000 nematodes per kg dry soil, V-high = >2000 nematodes per kg dry soil, *n* = number of samples and % = percentage of samples in which *Scottnema lindsayae* occurred. ^A^There may have been a typographical error in the original publication reporting this latitude/longitude. *Geographic coordinates associated with the recognized Antarctic place name for a general feature as listed by the USGS Advisory Committee on Antarctic Names (http://geonames.usgs.gov/antarctic/) and updated by the Polar Geospatial Center (http://www.pgc.umn.edu).

Biogeographic location	Lat, Long		Habitat	Abundance	Reference
**Victoria Land**	*74°15.00'S, 163°00.00'E				
-	NP, NP		“river”, wet mosses	Present	(Vinciguerra 1994)
**McMurdo Dry Valleys**	*77°30.00'S, 162°00.00'E				
-	NP, NP		soil	Present	(Freckman and Virginia 1990)
-	NP, NP		soil	M-high	(Freckman and Virginia 1993)
-	NP, NP		soil	Present	(Freckman and Virginia 1997)
***Alatna Valley***	*76°52.82'S, 161°13.82'E				
East, middle and south west end	NA, NA		soil	Medium (*n* = 20, 40%)	This paper, collected in 1995
Battleship Promontory	*76°54.85'S, 160°59.34'E				
-	NA, NA		soil	Medium (*n* = 17, 88%)	This paper, collected in 1993
-	76°55.30'S, 161°04.79'E		soil	M-high (*n* = 9, 22%)	This paper, collected in 1994
-	NA, NA		soil	Medium (*n* = 6, 83%)	This paper, collected in 1996
-	76°52.00'S, 161°05.00'E		soil	Present	(Courtright et al. 2000)
*Southwestern Bluff*	76°55.00'S, 161°03.00'E		soil	Medium (*n* = 14, 57%)	This paper, collected in 2001
-	76°55.30'S, 161°04.22'E		soil	Medium (*n* = 6, 83%)	This paper, collected in 2003
***Barwick Valley***	*77°20.71'S, 161°06.09'E		soil	Medium (*n* = 10, 40%)	This paper, collected in 1994
***Beacon Valley***	*77°49.00'S, 160°39.00'E		soil	Low (*n* = 24, 4%)	This paper, collected in 1990
***Garwood Valley***	*78°02.00'S, 164°10.00'E				
-	NA, NA		soil	High (*n* = 6, 100%)	This paper, collected in 1993
-	78°02.00'S, 164°10.00'E		soil	Medium	(Wall Freckman and Virginia 1998)
-	78°02.00'S, 164°10.00'E		soil	Present	(Courtright et al. 2000)
-	NA, NA		soil	V-high (*n* = 13, 100%)	This paper, collected in 2002
***McKelvey Valley***	*77°26.00'S, 161°33.00'E				
Upper	NA, NA		soil	High (*n* = 18, 50%)	This paper, collected in 1990
Lower	NA, NA		soil	Low (*n* = 14, 14%)	This paper, collected in 1990
***Miers Valley***	*78°06.00'S, 164°00.00'E				
Miers Glacier	*78°05.00'S, 163°40.00'E		moraine	Present	(Timm 1971)
-	NA, NA		soil	M-high (*n* = 24, 88%)	This paper, collected in 1990
***Taylor Valley***	*77°38.82'S, 163°03.08'E				
Canada Glacier	*77°37.00'S, 162°59.00'E		soil	Present	(Timm 1971)
Lacroix Glacier	*77°40.00'S, 162°33.00'E				
*Between Lake Bonney and Lacroix Glacier*	NP, NP		small runoff stream	Present	(Timm 1971)
*Southeast of Lacroix Glacier*	*77°40.00'S, 162°30.00'E		sandy soil	Present	(Timm 1971)
Lake Bonney	*77°43.00'S, 162°25.00'E				
*South side East Lobe*	77°42.92'S, 162°27.65'E		soil	Low (*n* = 9, 29%)	This paper, collected in 1993
	NA, NA		soil polygons	Medium (*n* = 99, 64%)	This paper, collected in 1994
-	NA, NA		soil	Medium (*n* = 2, 50%)	This paper, collected in 1995
-	77°42.92'S, 162°27.65'E		soil	Low	(Courtright et al. 1996)
-	NP, NP		soil polygons	Medium	(Moorhead et al. 1999)
*South side West Lobe*	77°42.5'S, 162°31.2'E		soil	Medium (*n* = 18, 94%)	This paper, collected in 1999, 2001 and 2002 (Simmons et al. 2009)
-	77°42.92'S, 162°27.65'E		soil	Present	(Courtright et al. 2000)
-	NA, NA		soil and stream sediment	Medium (*n* = 20, 45%)	This paper, collected in 2000
*South side West Lobe*	NA, NA		soil	M-low (*n* = 96, 45%)	This paper, collected in 2000, 2002 and 2003
*South side East Lobe*	77°42.55'S, 162°27.39'E		soil	Low	(Courtright et al. 2001)
Lake Chad	*77°38.55'S, 162°45.70'E		soil	Medium (*n* = 9, 22%)	This paper, collected in 1995
-	77°38.10'S, 162°48.15'E		soil	Present	(Boström et al. 2011)
Lake Fryxell	*77°36.58'S, 163°09.10'E				
-	NA, NA		soil	Medium (*n* = 26, 23%)	This paper, collected in 1990
*South side*	77°35.94'S, 163°22.68'E		soil	V-high (*n* = 9, 100%)	This paper, collected in 1993
-	77°35.94'S, 163°22.68'E		soil	High (*n* = 10, 80%)	This paper, collected in 1993
-	NA, NA		soil	High (*n* = 102, 87%)	This paper, collected in 1994
-	NA, NA		soil	Medium (*n* = 4, 75%)	This paper, collected in 1995
-	77°35.94'S, 163°22.68'E		soil	V-high	(Courtright et al. 1996)
*Von Guerard (F6) stream*	77°36.49'S, 163°14.95'E		soil	V-high (*n* = 30, 100%)	This paper, collected in 1996, 1997, 1998, 2001 and 2003
-	NA, NA		soil	V-high (*n* = 5, 100%)	This paper, collected in 1997
*Von Guerard Stream/ Harnish Creek network*	*77°37.00'S, 163°15.00'E		soil and stream sediments	Medium	(Treonis et al. 1999)
-	NP, NP		soil polygons	High	(Moorhead et al. 1999)
*Huey Creek stream*	*77°36.00'S, 163°06.00'E		soil	Medium (*n* = 7, 29%)	This paper, collected in 1999
*Von Guerard stream*	*77°37.00'S, 163°15.00'E		soil	High (*n* = 8, 100%)	This paper, collected in 1999
*South side F6 stream*	77°36.49'S, 163°14.92'E		soil	V-high (*n* = 12, 100%)	This paper, collected in 1999 and 2001
-	^A^77°55.94'S, 163°22.68'E		soil	Present	(Courtright et al. 2000)
*Harnish Creek transect*	*77°37.00'S, 163°13.00'E		soil and stream sediment	M-high (*n* = 20, 90%)	This paper, collected in 2000
*South side by F6 (SF)*	NA, NA		soil	V-high (*n* = 96, 100%)	This paper, collected in 2000, 2002 and 2003
*South-east shore*	77°35.56'S, 163°22.41'E		soil	V-high	(Courtright et al. 2001)
-	77°36.00'S, 162°15.00'E		soil	V-high	(Treonis et al. 2002)
*South side near F6*	77°36.40'S, 163°15.30'E		soil and lake sediment	High (*n* = 12, 67%)	This paper, collected in 2002
*South side near Green Creek*	77°37.36'S, 163°03.91'E		soil	M-high (*n* = 20, 85%)	This paper, collected in 2003
*South side near F6*	77°36.72'S, 163°15.18'E		soil	High (*n* = 20, 90%)	This paper, collected in 2003
*Von Guerard stream*	77°37.00'S, 163°15.00'E		soil	High	(Barrett et al. 2006c)
*Green Creek*	77°37.36'S, 163°03.91'E		soil	M-High	(Barrett et al. 2006c)
Lake Hoare	*77°38.00'S, 162°51.00'E				
*North side*	77°37.49'S, 162°54.31'E		soil	M-low (*n* = 18, 100%)	This paper, collected in 1993
*South side*	77°38.03'S, 162°52.75'E		soil	High (*n* = 9, 100%)	This paper, collected in 1993
*South side*	77°37.59'S, 162°52.57'E		soil	High (*n* = 56, 100%)	This paper, collected in 1993, 1994, 1995, 1996, 1997 and 2001
*North side*	77°38.00'S, 162°53.00'E		soil (0-2.5, 2.5-5, 5-10, 10-20 cm	High	(Powers et al. 1994a; 1995a)
*South shore*	NP, NP		soil at varying elevation	Medium	(Powers et al. 1998)
-	NA, NA		soil polygons	High (*n* = 104, 96%)	This paper, collected in 1994
*South side*	77°38.02'S, 162°52.23'E		soil	High (*n* = 40, 83%)	This paper, collected in 1994, 1995, 1996, 1997 and 2001
*North side*	77°38.00'S, 162°53.00'E		soil	Medium	(Powers et al. 1995a)
*South side*	77°38.00'S, 162°53.00'E		soil at varying elevation	M-high	(Powers et al. 1995a)
*South side*	77°38.00'S, 162°53.00'E		soil (0-2.5, 2.5-5, 5-10, 10-20 cm)	Medium	(Powers et al. 1995b)
*South side*	NA, NA		soil polygons	M-high (*n* = 24, 100%)	This paper, collected in 1995
*North side*	77°37.49'S, 162°54.31'E		soil	M-low	(Courtright et al. 1996)
*South side*	77°38.03'S, 162°52.75'E		soil	M-high	(Courtright et al. 1996)
-	NP, NP		soil	Medium	(Freckman and Virginia 1997)
*South side*	NA, NA		soil	M-high (*n* = 12, 100%)	This paper, collected in 1997
*South side*	77°38.00'S, 162°53.00'E		soil	Medium	(Powers et al. 1998)
-	NP, NP		soil polygons	High	(Moorhead et al. 1999)
*North side*	NA, NA		soil	V-high (*n* = 8, 100%)	This paper, collected in 1999
*South side*	NA, NA		soil	M-high (*n* = 8, 100%)	This paper, collected in 1999
*South side*	77°38.07'S, 162°52.59'E		soil	M-high (*n* = 18, 100%)	This paper, collected in 1999, 2001 and 2002
*North side*	77°37.49'S, 162°54.31'E		soil	Present	(Courtright et al. 2000)
*South side*	77°38.03'S, 162°52.75'E		soil	Present	(Courtright et al. 2000)
*South side*	77°38.00'S, 162°53.00'E		soil	M-high	(Treonis et al. 2000)
*North side*	77°37.29'S, 162°54.19'E		soil	M-low	(Courtright et al. 2001)
*South side*	77°38.02'S, 162°52.45'E		soil	M-high	(Courtright et al. 2001)
*South side*	77°38.00'S, 162°53.00'E		soil	M-high	(Porazinska et al. 2002b)
-	77°38.00'S, 162°53.00'E		soil	Medium	(Treonis et al. 2002)
-	77°37.90'S, 162°53.20'E		soil and lake sediments	M-high (*n* = 11, 73%)	This paper, collected in 2002
-	NP, NP		soil	Present	(Overhoff et al. 1993)
*North side*	NP, NP		lake bottom detritus	Present	(Vinciguerra 1994)
-	77°37.00'S, 160°50.00'E		soil	Medium	(Wall Freckman and Virginia 1998)
*South side*	NA, NA		wetlands (upland ponds)	M-low (*n* = 48, 19%)	This paper, collected in 2000
-	NP, NP		soil	High	(Treonis et al. 2000)
-	NP, NP		0-5 cm soil (exposed and subnivian)	High	(Gooseff et al. 2003)
*South side*	NP, NP		bare soil >80 m from wetlands (upland ponds)	Medium	(Moorhead et al. 2003)
Mummy Pond	77°40.06'S, 162°39.00'E		soil	Low (*n* = 5, 20%)	This paper, collected in 1997
Nussbaum Riegel	77°38.52'S, 162°46.89'E		soil	V-High (*n* = 5, 20%)	This paper, collected in 1997
***Victoria Valley***	*77°23.00'S, 162°00.00'E				
Lower Victoria Valley	77°21.81'S, 162°19.11'E		soil	High (*n* = 10, 60%)	This paper, collected in 1993
*NP*	77°21.81'S, 162°19.11'E		soil	High (*n* = 9, 100%)	This paper, collected in 1993
*Lake Vida*	*77°23.29'S, 161°56.05'E				
-	NA, NA		soil	Medium (*n* = 16, 19%)	This paper, collected in 1990
-	77°23.35'S, 162°02.60'E		soil	Medium (*n* = 10, 50%)	This paper, collected in 1993
-	NA, NA		soil	Medium (*n* = 11, 27%)	This paper, collected in 1994
-	77°22.58'S, 161°13.56'E		soil	NA (*n* = 2, 100%)	This paper, collected in 2000
*Vida Met Station*	NA, NA		soil	Low (*n* = 4, 50%)	This paper, collected in 2002
-	NA, NA		soil	M-high (*n* = 10, 80%)	This paper, collected in 1997
-	77°23.00'S, 162°00.00'E		soil	M-high	(Wall Freckman and Virginia 1998)
-	NA, NA		soil	NA (*n* = 6, 83%)	This paper, collected in 2003
*Victoria Lower Glacier*	*77°18.00'S, 162°40.00'E				
-	77°21.81'S, 162°19.11'E		soil	High	(Courtright et al. 1996)
-	77°22.57'S, 162°13.56'E		soil	NA (*n* = 6, 83%)	This paper, collected in 2000
-	77°21.81'S, 162°19.11'E		soil	Present	(Courtright et al. 2000)
*South-west*	77°21.49'S, 162°19.07'E		soil	High	(Courtright et al. 2001)
Victoria Upper Glacier	*77°16.00'S, 161°25.00'E				
-	77°17.35'S, 161°33.03'E		soil	High (*n* = 10, 60%)	This paper, collected in 1993
-	77°17.35'S, 161°33.03'E		soil	Low (*n* = 9, 11%)	This paper, collected in 1993
*Victoria Upper Lake*	*77°19.00'S, 161°35.00'E		soil	M-high (*n* = 20, 35%)	This paper, collected in 1990
***Wright Valley***	*77°31.39'S, 161°58.70'E				
Dais	*77°33.00'S, 161°16.00'E				
-	NP, NP		soil	Present	(Courtright et al. 2000)
-	NA, NA		soil	NA (*n* = 3, 100%)	This paper, collected in 2000
*East of Meserve Glacier*	*77°31.00'S, 162°17.00'E		algal mat	NP	(Timm 1971)
Labyrinth	*77°33.00'S, 160°50.00'E				
*West*	77°33.04'S, 160°43.15'E		soil	M-low (*n* = 9, 100%)	This paper, collected in 1993
-	77°33.04'S, 160°43.15'E		soil	M-low (*n* = 9, 78%)	This paper, collected in 1993
-	77°33.04'S, 160°43.15'E		soil	Low	(Courtright et al. 1996)
-	77°31.00'S, 161°50.00'E		soil	M-low	(Wall Freckman and Virginia 1998)
*West*	77°33.02'S, 160°43.09'E		soil	Low	(Courtright et al. 2001)
-	NA, NA		soil	Low (*n* = 12, 17%)	This paper, collected in 2003
Lake Brownworth	*77°26.00'S, 162°45.00'E				
-	NP, NP		soil	Present	(Overhoff et al. 1993)
-	77°26.13'S, 162°42.61'E		soil	M-low (*n* = 9, 33%)	This paper, collected in 1993
-	77°26.13'S, 162°42.61'E		soil	M-low	(Courtright et al. 1996)
-	77°26.13'S, 162°42.61'E		soil	Present	(Courtright et al. 2000)
*South-west shore*	77°26.08'S, 162°42.37'E		soil	M-low	(Courtright et al. 2001)
*Met Station*	NA, NA		soil	Medium (*n* = 4, 75%)	This paper, collected in 2002
Bull Pass	*77°28.00'S, 161°46.00'E		soil	Medium (*n* = 22, 23%)	This paper, collected in 1990
*Lake Bull*	*77°31.51'S, 161°42.68'E		soil	Low (*n* = 12, 17%)	This paper, collected in 1990
-	77°28.00'S, 161°46.00'E		soil	High (*n* = 24, 33%)	(Poage et al. 2008)
Lake Vanda	*77°32.00'S, 161°33.00'E				
*Near Lake Vanda*	77°32.00'S, 161°33.00'E		soil	Present	(Timm 1971)
*Vanda Station*	77°31.00'S, 161°40.00'E		soil	M-low (*n* = 2, 100%)	This paper, collected in 2002
Unspecified Locations					
-	NA, NA		soil	M-low (*n* = 5, 80%)	This paper, collected in 1997
-	NA, NA		soil	Present (*n* = 1, 100%)	This paper, collected in 2000
-	NA, NA		soil	Present (*n* = 10, 60%)	This paper, collected in 2003
**Koettlitz Glacier and Southern Coastal Regions**	*78°15.00'S, 164°15.00'E				
***Péwé Lake***	*77°56.67'S, 164°16.87'E		stony soil near the lake	Present	(Timm 1971)
***Strand Moraines***	*77°45.04'S, 164°29.90'E		sandy soil	Present	(Timm 1971)
***Marble Point***	*77°26.00'S, 163°50.00'E		mossy soil (*Bryum antarcticum*)	Present	(Timm 1971)
**Northern Coastal Region**					
***Cape Hallett***	72°19.29'S, 170°13.52'E		soil	Low (*n* = 67, 56%)	(Raymond et al. 2013a)
***Crater Cirque***	*72°37.49'S, 169°22.48'E		lake bottom detritus and wet mosses	Present	(Vinciguerra 1994)
***Edmonson Point***	*74°20.00'S, 165°08.00'E				
-	NA, NA		soil	Medium (*n* = 10, 80%)	This paper, collected in 1996
-	NA, NA		soil	Present (*n* = 28, 36%)	This paper, collected in 1996
-	NP, NP		soil	Present	(Bargagli et al. 1997)
-	NA, NA		soil	Medium (*n* = 8, 63%)	This paper, collected in 2001
***Gondwana Station***	74°37.57'S, 164°11.91'E		soil	M-Low (*n* = 371, 79%)	(Raymond et al. 2013a)
***Luther Peak***	*72°21.88'S, 169°50.91'E				
Luther Cirque	72°22.20'S, 169°53.10'E		soil	Medium (*n* = 40, 85%)	This paper, collected in 2003
Luther Vale North	72°22.00'S, 169°53.00'E		soil	Medium	(Barrett et al. 2006c)
Luther Vale South	72°22.00'S, 169°53.00'E		soil	Medium	(Barrett et al. 2006c)
***Terra Nova Bay***	*74°54.51'S, 164°27.19'E				
600 km north and south of the Italian station	NP, NP		mosses, lichens, fresh water sediments and penguin excrements (there are no details of whether *Scottnema lindsayae* occurred in all habitats or only in some)	Present	(Vinciguerra et al. 1994)
-	74°20.00'S, 165°08.00'E		soil	Present	(Courtright et al. 2000)

**Table 2.** Biogeographic distribution of *Plectus* species in Victoria Land, Antarctica. NP = not published, NA = not available, mur = *Plectus murrayi*. frig = *Plectus frigophilus*, where both exist = spp. For abundance, ^A^ abundance is per kg moss and adhering rock fragments not soil, Low = >0 to 20 nematodes per kg dry soil, M-low = 21 to 200 nematodes per kg dry soil, Medium = 201 to 600 nematodes per kg dry soil, M-high = 601 to 1000 nematodes per kg dry soil, High = 1001 to 2000 nematodes per kg dry soil, V-high = >2000 nematodes per kg dry soil, *n* = number of samples and % = percentage of samples in which *Plectus* occurred. For references to “this paper”, the year collected refers to the year at the beginning of the austral summer in which samples were collected to 0-10 cm depth. ^B^This publication refers to a map for more details on sample location.

Biogeographic location	Lat, Long		Habitat	Species	Abundance	Reference
**McMurdo Dry Valleys**	*77°30.00'S, 162°00.00'E					
-	NP, NP		soil	spp.	Present	(Freckman and Virginia 1990)
-	NP, NP		soil	spp.	Low	(Freckman and Virginia 1993)
-	NP, NP		soil	*mur*	Present	(Freckman and Virginia 1997)
-	NP, NP		streams	spp.	Present	(Moorhead et al. 1999)
***Alatna Valley***	*76°52.82'S, 161°13.82'E					
Battleship Promontory	*76°54.85'S, 160°59.34'E		soil	*mur*	Low (*n* = 17, 6%)	This paper, collected in 1993
*East, middle and southwestern end*	NA, NA		soil	*mur*	Low (*n* = 20, 10%)	This paper, collected in 1995
***Garwood Valley***	*78°02.00'S, 164°10.00'E					
Garwood Lake	*78°02.00'S, 164°15.00'E		NP	*frig*	Present	(Timm 1971)
-	NA, NA		soil	*mur*	M-low (*n* = 6, 50%)	This paper, collected in 1993
-	78°02.00'S, 164°10.00'E		soil	*mur*	M-low	(Wall Freckman and Virginia 1998)
-	NA, NA		soil	*mur*	Low (*n* = 13, 8%)	This paper, collected in 2002
***Miers Valley***	*78°06.00'S, 164°00.00'E					
Miers Glacier	*78°05.00'S, 163°40.00'E		mossy soil from glacier foot, runoff stream	*frig*	Present	(Timm 1971)
-	NA, NA		soil	*mur*	M-low (*n* = 24, 29%)	This paper, collected in 1990
***Taylor Valley***	*77°38.82'S, 163°03.08'E					
Canada Glacier	*77°37.00'S, 162°59.00'E					
*Near the glacier*	NP, NP		soil	*frig*	Present	(Timm 1971)
-	77°37.31'S, 162°58.26'E		windblown sediment on top of glacier	*mur*	Present (*n* = 2, 100%)	This paper, collected in 1997
*Waterfall (upper west)*	NA, NA		cryconite hole	*mur*	Present	This paper, collected in 2001
Lake Bonney	*77°43.00'S, 162°25.00'E					
-	NP, NP		lake, soil nearby	*frig*	Present	(Timm 1971)
-	NA, NA		soil polygon cracks	*mur*	Low (*n* = 99, 5%)	This paper, collected in 1994
-	NA, NA		soil	*mur*	Medium (*n* = 2, 100%)	This paper, collected in 1995
-	NA, NA		algal mat	spp.	Present (*n* = 5, 100%)	This paper, collected in 1995
*West Lobe*	77°43.50'S, 162°18.95'E		soil	*mur*	Low (*n* = 18, 33%)	This paper, collected in 1999, 2001 and 2002
-	NA, NA		soil and stream sediment	*mur*	M-low (*n* = 20, 30%)	This paper, collected in 2000
*West Lobe*	NA, NA		soil	*mur*	Low (*n* = 72, 7%)	This paper, collected in 2000 and 2003
-	77°43.40'S, 162°18.40'E		soil and sediment	*mur*	Low (*n* = 12, 25%)	This paper, collected in 2002
Lake Chad	*77°38.55'S, 162°45.70'E					
-	NP, NP		NP	*frig*	Present	(Timm 1971)
-	NA, NA		algal mat	spp.	NA (*n* = 1, 100%)	This paper, collected in 1995
-	NA, NA		soil	*mur*	M-low (*n* = 9, 56%)	This paper, collected in 1995
Lake Fryxell	*77°36.58'S, 163°09.10'E					
-	NP, NP		NP	*frig*	Present	(Timm 1971)
-	NP, NP		algae in a drift stream near the lake	spp.	Present	(Wharton and Brown 1989)
-	NA, NA		algal mat	*ant*	M-low (*n* = 10, 100%)	This paper, collected in 1990
-	NA, NA		soil	*mur*	M-high (*n* = 26, 77%)	This paper, collected in 1990
-	77°35.94'S, 163°22.68'E		soil	*mur*	Low (*n* = 10, 10%)	This paper, collected in 1993
-	NA, NA		algal mat	spp.	NA (*n* = 1, 100%)	This paper, collected in 1995
-	NA, NA		soil	*mur*	Medium (*n* = 4, 75%)	This paper, collected in 1995
*Von Guerard stream / Harnish Creek network*	*77°37.00'S, 163°15.00'E		stream sediments and surrounding soils	spp.	M-low	(Treonis et al. 1999)
*Huey Creek*	*77°36.00'S, 163°06.00'E		soil	*mur*	M-low (*n* = 7, 57%)	This paper, collected in 1999
*Harnish Creek*	*77°37.00'S, 163°13.00'E		soil and sediment	*mur*	M-low (*n* = 20, 60%)	This paper, collected in 2000
*South side*	NA, NA		soil	*mur*	Low (*n* = 72, 4%)	This paper, collected in 2000 and 2002
*South side*	77°36.40'S, 163°15.30'E		soil and sediment	*mur*	V-High (*n* = 12, 75%)	This paper, collected in 2002
*South side*	77°36.49'S, 163°14.95'E		soil	*mur*	Low (*n* = 6, 17%)	This paper, collected in 2003
*South side*	77°36.49'S, 163°14.92'E		soil	*mur*	Low (*n* = 6, 17%)	This paper, collected in 2003
*South side near Green Creek*	77°37.36'S, 163°03.91'E		soil	*mur*	Medium (*n* = 20, 60%)	This paper, collected in 2003
*Green Creek*	77°37.36'S, 163°03.91'E		soil	*mur*	Medium	(Barrett et al. 2006c)
Lake Hoare	*77°38.00'S, 162°51.00'E					
*North side*	77°37.49'S, 162°54.31'E		soil	*mur*	Low (*n* = 18, 6%)	This paper, collected in 1993
*South side*	NP, NP		soil at varying elevation	spp.	Low	(Powers et al. 1994b; 1998)
*South side*	77°38.00'S, 162°53.00'E		soil (0-2.5, 2.5-5, 5-10, 10-20 cm)	*mur*	Low	(Powers et al. 1995a)
*South side*	77°37.93'S, 162°53.19'E		soil at varying elevation	*mur*	Low (*n* = 150, 6%)	This paper, collected in 1995, 1998 and 2002
*North side*	77°37.49'S, 162°54.31'E		soil	*mur*	Low	(Courtright et al. 1996)
*South side*	77°38.00'S, 162°53.00'E		soil	mur	Low	(Powers et al. 1998)
*North side*	NA, NA		soil	*mur*	M-low (*n* = 8, 50%)	This paper, collected in 1999
*North side*	77°37.29'S, 162°54.19'E		soil	*mur*	Low	(Courtright et al. 2001)
*South side*	77°38.02'S, 162°52.23'E		soil	*mur*	Low (*n* = 8, 13%)	This paper, collected in 2001
*South side*	77°38.00'S, 162°53.00'E		soil	*mur*	Low	(Porazinska et al. 2002b)
-	77°37.90'S, 162°53.20'E		soil and lake sediment	*mur*	V-High (*n* = 11, 82%)	This paper, collected in 2002
*South side*	77°38.02'S, 162°53.05'E		soil	*mur*	Low (*n* = 6, 17%)	This paper, collected in 2003
-	77°37.00'S, 162°50.00'E		soil	*mur*	M-low	(Wall Freckman and Virginia 1998)
-	NP, NP		soil	*mur*	M-low	(Treonis et al. 2000)
*South side*	NA, NA		high elevation upland pond areas	spp.	Low (*n* = 48, 19%)	This paper, collected in 2000
-	NP, NP		0-5 cm soil (subnivian)	*mur*	M-low	(Gooseff et al. 2003)
*South side*	NP, NP		high elevation upland pond areas	spp.	M-low	(Moorhead et al. 2003)
Taylor Glacier	*77°44.00'S, 162°10.00'E		windblown sediment on top of glacier	*mur*	Present (*n* = 1, 100%)	This paper, collected in 1998
Suess Glacier	*77°38.00'S, 162°40.00'E		soil nearby	*frig*	Present	(Timm 1971)
Suess Lake	NP, NP		NP	*frig*	Present	(Timm 1971)
***Victoria Valley***	*77°23.00'S, 162°00.00'E		soil	*mur*	Present (*n* = 6, 17%)	This paper, collected in 2003
***Wright Valley***	*77°31.39'S, 161°58.70'E					
Along Onyx River	*77°31.31'S, 161°49.39'E		pond	spp.	Present	(Timm 1971)
East of Meserve Glacier	*77°31.00'S, 162°17.00'E		algal mat	spp.	Present	(Timm 1971)
Canopus Pond	NP, NP		NP	*frig*	Present	(Timm 1971)
Lake Vanda	*77°32.00'S, 161°33.00'E		lake, soil nearby	*frig*	Present	(Timm 1971)
Lower Wright Lake (=Lake Brownworth)	*77°26.00'S, 162°45.00'E		NP	*frig*	Present	(Timm 1971)
Edge of Lake Canopus	*77°33.00'S, 161°31.00'E		algal growth at the edge of the lake	spp.	Present	(Wharton and Brown 1989)
Between Lake Vanda and Lake Bull	NP, NP		dry algae around the edge of small ponds	spp.	Present	(Wharton and Brown 1989)
Between Lake Vanda and Lake Bull	NP, NP		wet algae in meltwater and around the edge of small ponds	spp.	Present	(Wharton and Brown 1989)
Bull Pass	*77°28.00'S, 161°46.00'E		soil	*mur*	M-low (*n* = 22, 18%)	This paper, collected in 1990
*Lake Bull*	*77°31.51'S, 161°42.68'E		soil	*mur*	Low (*n* = 12, 8%)	This paper, collected in 1990
-	77°31.00'S, 161°50.00'E		soil	*mur*	M-low	(Wall Freckman and Virginia 1998)
**Koettlitz Glacier and Southern Coastal Regions**	*78°15.00'S, 164°15.00'E					
***Cape Chocolate*** (just north of)	*77°56.05'S, 164°34.70'E		moraine	*frig*	Present	(Timm 1971)
***Marble Point***	*77°26.00'S, 163°50.00'E					
-	NP, NP		moss (*Bryum antarcticum*)	*mur*	V-high^A^	(Yeates 1970)
-	NP, NP		mossy soil and melt pools with abundant algae (*Nostoc commune*)	*mur*	Present	(Timm 1971)
-	NP, NP		meltpools w/ abundant algae (*Nostoc commune*), mossy soil	*frig*	Present	(Timm 1971)
***Pewe Lake***	NP, NP		NP	*frig*	Present	(Timm 1971)
***Strand Moraines***	*77°45.04'S, 164°29.90'E					
-	NP, NP		mossy soil and melt pools with abundant algae (*Nostoc commune*)	spp.	Present	(Timm 1971)
-	NP, NP		sandy soil, mossy soil, stream with abundant algae	*frig*	Present	(Timm 1971)
**Northern Coastal Region**						
***Cape Hallett***	*72°19.00'S, 170°16.00'E					
-	NP, NP		NP	spp.	Present	(Timm 1971)
Willett Cove	72°19.00'S, 170°14.00'E		soil	*mur*	Medium	(Barrett et al. 2006c)
-	NA, NA		soil amongst penguin rookery	*mur*	M-low (*n* = 20, 30%)	This paper, collected in 2003
-	72°19.29'S, 170°13.52'E		soil		Low (*n* = 67, 56%)	(Raymond et al. 2013a)
***Edmonson Point***	*74°20.00'S, 165°08.00'E					
-^B^	NP, NP		wet moss near a brook	*mur*	Present	(Vinciguerra 1994)
-	NP, NP		soil	spp.	Present	(Bargagli et al. 1997)
-	NA, NA		soil	*mur*	M-high (*n* = 10, 70%)	This paper, collected in 1996
-	NA, NA		soil	*mur*	NA (*n* = 28, 50%)	This paper, collected in 1996
-	NA, NA		soil	*mur*	M-low (*n* = 8, 63%)	This paper, collected in 2001
***Gondwana Station***	74°37.57'S, 164°11.91'E		soil		Low (*n* = 371, 84%)	(Raymond et al. 2013a)
***Luther Peak***	72°22.20'S, 169°53.10'E		soil	*mur*	Low (*n* = 40, 8%)	This paper, collected in 2003
Luther Vale South	72°22.00'S, 169°53.00'E		soil	*mur*	Low	(Barrett et al. 2006c)
***Terra Nova Bay***	*74°54.51'S, 164°27.19'E					
600 km north and south of the Italian station	NP, NP		mosses, lichens, fresh-water sediments and penguin excrements (no details of whether *Plectus* occurred in all habitats or only in some)	spp.	Present	(Vinciguerra et al. 1994)
***Barclay Glacier***						
-	NP, NP		algae growing in meltwater	*mur*	Present	(Wharton and Brown 1989)

**Table 3.** Biogeographic distribution of *Eudorylaimus* species in Victoria Land, Antarctica. NP = not published. NA = not available. *ant* = *Eudorylaimus antarcticus*. *gla* = *Eudorylaimus glacialis*. For abundance, Low = >0 to 20 nematodes per kg dry soil, M-low = 21 to 200 nematodes per kg dry soil, Medium = 201 to 600 nematodes per kg dry soil, M-high = 601 to 1000 nematodes per kg dry soil, High = 1001 to 2000 nematodes per kg dry soil, V-high = >2000 nematodes per kg dry soil, *n* = number of samples and % = percentage of samples in which *Eudorylaimus* occurred. ^A^There may have been a typographical error in the original publication reporting this longitude. ^B^ASPA = Antarctic Specially Protected Area (previously Site of Special Scientific Interest). For references to “this paper”, the year collected refers to the year at the beginning of the austral summer in which samples were collected at 0-10 cm depth.

Biogeographic location	Lat, Long		Habitat	Species	Abundance	Reference
**McMurdo Dry Valleys**	*77°30.00'S, 162°00.00'E					
-	NP, NP		soil	*ant*	Present	(Freckman and Virginia 1990)
-	NP, NP		soil	*ant*, *gla*	M-low	(Freckman and Virginia 1993)
-	NP, NP		soil	*ant*	Present	(Freckman and Virginia 1997)
-	NP, NP		soil, sediment	*ant*	Present	(Moorhead et al. 1999)
***Alatna Valley***	*76°52.82'S, 161°13.82'E					
Battleship Promontory	*76°54.85'S, 160°59.34'E					
-	NA, NA		soil	*ant*	M-low (*n* = 17, 65%)	This paper, collected in 1993
-	76°55.30'S, 161°04.79'E		moist soil, green with algae and between dolomite rocks	*ant*	Low (*n* = 9, 22%)	This paper, collected in 1994
-	NA, NA		soil	*ant*	M-low (*n* = 6, 17%)	This paper, collected in 1996
*Southwestern Bluff*	76°55.00'S, 161°03.00'E		soil	*ant*	Low (*n* = 14, 7%)	This paper, collected in 2001
-	NA, NA		soil	*ant*	Low (*n* = 6, 50%)	This paper, collected in 2003
***Garwood Valley***	*78°02.00'S, 164°10.00'E					
Garwood Lake	*78°01.58'S, 164°15.42'E		NP	*ant*	Present	(Timm 1971)
-	NA, NA		soil	*ant*	M-low (*n* = 6, 100%)	This paper, collected in 1993
-	78°02.00'S, 164°10.00'E		soil	*ant*	M-low	(Wall Freckman and Virginia 1998)
-	NA, NA		soil	*ant*	M-low (*n* = 13, 23%)	This paper, collected in 2002
***McKelvey Valley***	*77°26.00'S, 161°33.00'E					
Upper	NA, NA		soil	*ant*	M-low (*n* = 18, 33%)	This paper, collected in 1990
***Miers Valley***	*78°06.00'S, 164°00.00'E					
Miers Glacier (the foot of)	*78°05.00'S, 163°40.00'E		moss	*ant*	Present	(Timm 1971)
*Runoff stream from the Miers Glacier*	*78°05.00'S, 163°40.00'E		NP	*ant*	Present	(Timm 1971)
Miers Lake	*78°06.00'S, 163°51.00'E		NP	*ant*	Present	(Timm 1971)
-	NA, NA		soil	*ant*	M-low (*n* = 24, 50%)	This paper, collected in 1990
***Taylor Valley***	*77°38.82'S, 163°03.08'E					
Lake Bonney	*77°43.00'S, 162°25.00'E					
-	NP, NP		NP	*ant*	Present	(Timm 1971)
-	NA, NA		soil	*ant*	Low (*n* = 99, 52%)	This paper, collected in 1994
-	NA, NA		soil	*ant*	M-low (*n* = 2, 100%)	This paper, collected in 1995
-	NA, NA		algal mat	*ant*	NA (*n* = 5, 60%)	This paper, collected in 1995
-	NP, NP		soil, sediment	*ant*	Low	(Moorhead et al. 1999)
*West Lobe*	77°43.50'S, 162°18.95'E		soil	*ant*	Low (*n* = 18, 61%)	This paper, collected in 1999, 2001 and 2002
-	NA, NA		soil, sediment	*ant*	M-low (*n* = 20, 35%)	This paper, collected in 2000
*West Lobe*	NA, NA		soil	*ant*	Low (*n* = 48, 2%)	This paper, collected in 2000
Lake Chad	*77°38.55'S, 162°45.70'E					
-	NP, NP		NP	*ant*	Present	(Timm 1971)
-	NA, NA		soil	*ant*	M-low (*n* = 9, 56%)	This paper, collected in 1995
-	NA, NA		algal mat	*ant*	NA (*n* = 1, 100%)	This paper, collected in 1995
Lake Fryxell	*77°36.58'S, 163°09.10'E					
-	NP, NP		NP	*ant*	Present	(Timm 1971)
-	NP, NP		algae in a drift stream near the lake	*ant*	Present	(Wharton and Brown 1989)
-	NA, NA		plant material	*ant*	Present (*n* = 10, 100%)	This paper, collected in 1990
-	NA, NA		soil	*ant*	Medium (*n* = 26, 77%)	This paper, collected in 1990
*South side*	77°35.94'S, 163°22.68'E		soil	*ant*	Low (*n* = 9, 11%)	This paper, collected in 1993
-	NA, NA		soil	*ant*	Low (*n* = 102, 41%)	This paper, collected in 1994
*South side*	77°36.49'S, 163°18.95'E		soil	*ant*	Low (*n* = 18, 33%)	This paper, collected in 1996, 1998 and 2001
-	NA, NA		soil	*ant*	Low (*n* = 5, 20%)	This paper, collected in 1997
-	NA, NA		algal mat	*ant*	Present (*n* = 1, 100%)	This paper, collected in 1998
-	NA, NA		soil	*ant*	Medium (*n* = 4, 75%)	This paper, collected in 1998
*Von Guerard stream/ Harnish Creek*	*77°37.00'S, 163°15.00'E		stream sediments and surrounding soils	*ant*, *gla*	Medium	(Treonis et al. 1999)
-	NP, NP		soil, sediment	*ant*	Low	(Moorhead et al. 1999)
*Von Guerard stream*	*77°37.00'S, 163°15.00'E		soil	*ant*	M-low (*n* = 8, 63%)	This paper, collected in 1999
*Huey Creek stream*	*77°36.00'S, 163°06.00'E		soil	*ant*	M-low (*n* = 7, 29%)	This paper, collected in 1999
*South side*	77°36.49'S, 163°14.92'E		soil	*ant*	M-low (*n* = 12, 83%)	This paper, collected in 1999 and 2001
*Harnish Creek*	*77°37.00'S, 163°13.00'E		soil and stream sediment	*ant*	Medium (*n* = 20, 70%)	This paper, collected in 2000
*South side*	NA, NA		soil	*ant*	M-low (*n* = 96, 97%)	This paper, collected in 2000, 2002 and 2003
-	77°36.00'S, 162°15.00'E		soil	*ant*	Low	(Treonis et al. 2002)
*South side near F6 stream*	77°36.40'S, 163°15.30'E		soil and lake sediment	*ant*	M-low (*n* = 12, 33%)	This paper, collected in 2002
*South side near Green Creek*	77°37.36'S, 163°03.91'E		soil	*ant*	Medium (*n* = 20, 45%)	This paper, collected in 2003
*South Side near F6 stream*	77°36.72'S, 163°15.18'E		soil	*ant*	M-low (*n* = 20, 35%)	This paper, collected in 2003
*Green Creek*	77°37.36'S, 163°03.91'E		soil	?	Medium	(Barrett et al. 2006c)
*Von Guerard stream*	*77°37.00'S, 163°15.00'E		soil	?	M-Low	(Barrett et al. 2006c)
Lake Hoare	*77°38.00'S, 162°51.00'E					
*North side*	77°37.49'S, 162°54.31'E		soil	*ant*	Low (*n* = 18, 78%)	This paper, collected in 1993
*South side*	77°38.03'S, 162°52.75'E		soil	*ant*	Low (*n* = 9, 33%)	This paper, collected in 1993
*South side*	NA, NA		soil	*ant*	Low (*n* = 12, 25%)	This paper, collected in 1993
*South side*	77°37.59'S, 162°52.57'E		soil	*ant*	M-low (*n* = 56, 77%)	This paper, collected in 1993, 1994, 1995, 1996, 1997 and 2001
*North side*	77°38.00'S, 162°53.00'E		soil (0-2.5, 2.5-5, 5-10, 10-20 cm)	*ant*	M-low	(Powers et al. 1994a)
*South side*	NP, NP		soil at varying elevations	*ant*, *gla*	M-low	(Powers et al. 1994b)
-	NA, NA		soil polygons	*ant*	Low (*n* = 104, 17%)	This paper, collected in 1994
*North side*	77°38.00'S, 162°53.00'E		soil (0-2.5, 2.5-5, 5-10, 10-20 cm)	*ant*	M-low	(Powers et al. 1995b)
*South side*	77°38.00'S, 162°53.00'E		soil at varying elevations	*ant*	Low	(Powers et al. 1995a)
*South side*	NA, NA		soil polygons	*ant*	Low (*n* = 24, 54%)	This paper, collected in 1995
*South side*	77°37.93'S, 162°53.19'E		soil	*ant*	M-low (*n* = 150, 51%)	This paper, collected in 1995, 1998 and 2002
*North side*	77°37.49'S, 162°54.31'E		soil	*ant*	Low	(Courtright et al. 1996)
-	NP, NP		soil	*ant*	M-low	(Freckman and Virginia 1997)
*South side*	77°38.00'S, 162°53.00'E		soil	*ant*, *gla*	Medium	(Powers et al. 1998)
-	NP, NP		soil, sediment	*ant*	Low	(Moorhead et al. 1999)
*North side*	NA, NA		soil	*ant*	Low (*n* = 8, 38%)	This paper, collected in 1999
*South side*	NA, NA		soil	*ant*	M-low (*n* = 8, 75%)	This paper, collected in 1999
*South side*	77°38.00'S, 162°53.00'E		soil	*ant*	M-low	(Treonis et al. 2000; 2002)
*North side*	77°37.29'S, 162°54.19'E		soil	*ant*	Low	(Courtright et al. 2001)
*South side*	77°38.07'S, 162°52.59'E		soil	*ant*	Low (*n* = 12, 58%)	This paper, collected in 2001, 2002
*South side*	77°38.00'S, 162°53.00'E		soil	*ant*	M-low	(Porazinska et al. 2002b)
-	77°37.90'S, 162°53.20'E		soil and lake sediment	*ant*	M-low (*n* = 11, 64%)	This paper, collected in 2002
-	77°37.00'S, 160°50.00'E		soil	*ant*	M-low	(Wall Freckman and Virginia 1998)
-	NP, NP		soil	*ant*	M-low	(Treonis et al. 2000)
-	NP, NP		0-5 cm soil (subnivian)	*ant*	M-low	(Gooseff et al. 2003)
Nussbaum Riegel	77°38.52'S, 162°46.89'E		soil	*ant*	Low (*n* = 5, 60%)	This paper, collected in 1997
Suess Glacier, 50 m away	*77°38.00'S, 162°40.00'E		soil	*ant*	Present	(Timm 1971)
Suess Pond	NP, NP		NP	*ant*	Present	(Timm 1971)
***Victoria Valley***	*77°23.00'S, 162°00.00'E					
Lake Vida	*77°23.29'S, 161°56.05'E		NP	*ant*	Present	(Timm 1971)
Upper	NA, NA		soil	*ant*	Low (*n* = 20, 5%)	This paper, collected in 1990
Victoria Upper Glacier	77°17.35'S, 161°33.03'E		soil	*ant*	Low (*n* = 10, 43%)	This paper, collected in 1993
-	77°23.00'S, 162°00.00'E		soil	*ant*	Low	(Wall Freckman and Virginia 1998)
-	NA, NA		soil	*ant*	Present (*n* = 6, 50%)	This paper, collected in 2003
***Wright Valley***	*77°31.39'S, 161°58.70'E					
Between Lake Vanda and Lake Bull	NP, NP		dry algae around the edge of small ponds	*ant*	Present	(Wharton and Brown 1989)
Dais	*77°33.00'S, 161°16.00'E		soil	*ant*	Low (*n* = 3, 67%)	This paper, collected in 2000
East of Meserve Glacier	*77°31.00'S, 162°17.00'E		algal mat	*ant*	Present	(Timm 1971)
Labyrinth	*77°33.00'S, 160°50.00'E					
*West*	77°33.04'S, 160°43.15'E		soil	*ant*	Low (*n* = 9, 89%)	This paper, collected in 1993
-	77°33.04'S, 160°43.15'E		soil	*ant*	Low (*n* = 9, 11%)	This paper, collected in 1993
-	77°33.04'S, 160°43.15'E		soil	*ant*	Low	(Courtright et al. 1996)
*West*	77°33.02'S, 160°43.09'E		soil	*ant*	Low	(Courtright et al. 2001)
-	NA, NA		soil	*ant*	Low (*n* = 12, 8%)	This paper, collected in 2003
Bull Pass	*77°28.00'S, 161°46.00'E		soil	*ant*	Low (*n* = 12, 33%)	This paper, collected in 1990
*Bull Lake*	*77°31.51'S, 161°42.68'E		soil	*ant*	Low (*n* = 22, 5%)	This paper, collected in 1990
-	*77°28.00'S, 161°46.00'E		soil	*ant*	M-Low (*n* = 24, 12.5%)	(Poage et al. 2008)
Lake Vanda	*77°32.00'S, 161°33.00'E					
-	NP, NP		NP	*ant*	Present	(Timm 1971)
*Near Lake Vanda and Péwé Lake*	*77°32.00'S, 161°33.00'E		stony soil	*ant*	Present	(Timm 1971)
*Met Station*	NA, NA		soil	*ant*	M-low (*n* = 2, 100%)	This paper, collected in 2002
Lake Brownworth	*77°26.00'S, 162°45.00'E		NP	*ant*	Present	(Timm 1971)
-	NA, NA		soil	*ant*	Low (*n* = 5, 60%)	This paper, collected in 1997
-	77°31.00'S, 161°50.00'E		soil	*ant*	M-low	(Wall Freckman and Virginia 1998)
-	NA, NA		soil	*ant*	Present (*n* = 10, 50%)	This paper, collected in 2003
Onyx River pond	*77°32.00'S, 161°45.00'E		NP	*ant*	Present	(Timm 1971)
Linnaeus Terrace ASPA^B^	77°35.83'S, 161°05.00'E		soil	*ant*	Low (*n* = 16, 6%)	This paper, collected in 1990
**Koettlitz Glacier and Southern Coastal Regions**	*78°15.00'S, 164°15.00'E					
***Cape Chocolate***(north)	*76°56.00'S, 164°35.00'E		moraine	*ant*	Present	(Timm 1971)
***Strand Moraines***	*77°45.04'S, 164°29.90'E		algal mat (in stream bed), sandy soil	*ant*	Present	(Timm 1971)
***Marble Point***	*77°26.00'S, 163°50.00'E		NP	*ant*	Present	(Timm 1971)
**Northern Coastal Regions**						
***Cape Adare***	*71°17.00'S, 170°14.00'E		NP	*ant*	Present	(Timm 1971)
***Cape Hallett***	*72°19.00'S, 170°16.00'E					
Hallett Station	*72°19.00'S, 170°16.00'E		NP	*ant*	Present	(Timm 1971)
-	NA, NA		soil	*ant*	Low (*n* = 20, 20%)	This paper, collected in 2003
Cape Hallett	72°19.29'S, 170°13.52'E		soil		Low (*n* = 67, 67%)	(Raymond et al. 2013a)
***Edmonson Point***	*74°20.00'S, 165°08.00'E					
-	NA, NA		soil	*ant*	Low (*n* = 10, 30%)	This paper, collected in 1996
-	NA, NA		soil	*ant*	Present (*n* = 28, 7%)	This paper, collected in 1996
-	NP, NP		NP	*ant*, *gla*	Present	(Bargagli et al. 1997)
-	NA, NA		soil	*ant*	Low (*n* = 8, 25%)	This paper, collected in 2001
***Gondwana Station***	74°37.57'S, 164°11.91'E		soil	*ant*	Low (*n* = 371, 37%)	(Raymond et al. 2013a)
***Luther Peak***	*72°21.88'S, 169°50.91'E		soil	*ant*	M-low (*n* = 40, 85%)	This paper, collected in 2003
Luther Vale North	72°22.00'S, 169°53.00'E		soil	?	M-Low	(Barrett et al. 2006c)
Luther Vale South	72°22.00'S, 169°53.00'E		soil	?	M-Low	(Barrett et al. 2006c)
***Terra Nova Bay***	*74°54.51'S, 164°27.19'E					
600 km north and south of the Italian station	NP, NP		mosses, lichens, fresh-water sediments and penguin excrement (there are no details of whether *Eudorylaimus* occurred in all habitats or only in some)	*ant*	Present	(Vinciguerra et al. 1994)
***Barclay Glacier***	NP, NP		algae in meltwater	*ant*	Present	(Wharton and Brown 1989)

**Table 4.** Biogeographic distribution of *Panagrolaimus davidi* in Victoria Land, Antarctica. NP = not published. NA = not available. For references to “this paper”, the year collected refers to the year at the beginning of the austral summer in which samples were collected. For abundance, M-low = 21 to 200 nematodes per kg dry soil, Medium = 201 to 600 nematodes per kg dry soil, *n* = number of samples and % = percentage of samples in which *Panagrolaimus* occurred.

Biogeographic location	Lat, Long		Habitat	Abundance	Reference
**McMurdo Dry Valleys**	*77°30.00'S, 162°00.00'E				
***Miers Valley***	*78°06.00'S, 164°00.00'E		soil	M-low (*n* = 24, 29%)	This paper, collected in 1990
**Southern Coastal Region**					
***Marble Point***	*77°26.00'S, 163°50.00'E		mossy soil (*Bryum antarcticum*)	Present	(Timm 1971)
**Northern Coastal Region**					
***Cape Bird***	77°13.00'S, 166°26.00'E		soil in penguin rookery	Medium (*n* = 29, 52%)	(Porazinska et al. 2002a)
***Cape Crozier***	77°27.00'S, 169°11.00'E		soil in penguin rookery	M-low (*n* = 27, 48%)	(Porazinska et al. 2002a)
***Cape Hallett***	*72°19.00'S, 170°16.00'E				
-	NA, NA		soil in penguin rookery	Low (*n* = 2, 50%)	This paper, collected in 2002
Willet Cove	72°19.00'S, 170°14.00'E		soil	M-High	(Barrett et al. 2006c)
Seabee Spit	72°18.83'S, 170°13.00'E		soil	Low	(Barrett et al. 2006c)
Cape Hallett	72°19.29'S, 170°13.52'E		soil	M-Low (*n* = 56, 56%)	(Raymond et al. 2013a)
***Cape Royds***	77°33.00'S, 166°10.00'E		soil amongst penguin rookery	M-low (*n* = 66, 20%)	(Porazinska et al. 2002a)
-	NA, NA		soil pits amongst penguin rookery	Med (*n* = 20, 70%)	This paper, collected in 2003
***Edmonson Point***	*74°20.00'S, 165°08.00'E		soil	Present (*n* = 28, 4%)	This paper, collected in 1996
***Gondwana Station***	74°37.57'S, 164°11.91'E		soil	M-Low (*n* = 371, 34%)	(Raymond et al. 2013a)

**Table 5.** Biogeographic distribution of *Geomonhystera antarcticola* in Victoria Land, Antarctica. NP = not published. NA = not available. For references to “this paper”, the year collected refers to the year at the beginning of the austral summer in which samples were collected. For abundance, Low = >0 to 20 nematodes per kg dry soil, M-low = 21 to 200 nematodes per kg dry soil, *n* = number of samples and % = percentage of samples in which *Geomonhystera* occurred.

Biogeographic location	Lat, Long		Habitat	Abundance	Reference
**McMurdo Dry Valleys**	*77°30.00'S, 162°00.00'E				
***Alatna Valley***	*76°52.82'S, 161°13.82'E				
Battleship Promontory	*76°54.85'S, 160°59.34'E				
-	NA, NA		soil	Low (*n* = 17, 47%)	This paper, collected in 1993
*Southwestern Bluff*	76°55.00'S, 161°03'.00E		soil	Low (*n* = 14, 14%)	This paper, collected in 2001
-	NA, NA		soil	Low (*n* = 6, 50%)	This paper, collected in 2003
***Taylor Valley***	*77°38.82'S, 163°03.08'E				
Lake Bonney	*77°43.00'S, 162°25.00'E		soil	M-low (*n* = 2, 50%)	This paper, collected in 1998
***Wright Valley***	*77°31.39'S, 161°58.70'E				
183 m east of Meserve Glacier	*77°31.00'S, 162°17.00'E		algal mat on soil	Present	(Timm 1971)
Between Lake Vanda and Lake Bull	NP, NP		dry algae from the edge of a small pond	Present	(Wharton and Brown 1989)
-			soil	Low (*n* = 10, 20%)	This paper, collected in 2003
**Northern Coastal Region**					
***Edmonson Point***	*74°20.00'S, 165°08.00'E		soil	Present	(Bargagli et al. 1997)

**Table 6.** Ecology of Nematode Genera in Victoria Land.

Genus	Co-occurs with	Nematode community complexity	Feeding	Reproduction
*Scottnema*	*Eudorylaimus*, *Plectus*, *Geomonhystera*, *Panagrolaimus* (rare, only in Dry Valleys; Bargagli et al. 1997; Courtright et al. 2001; this paper),	1 species- most common 2 species- often (usually *Eudorylaimus antarcticus*), 3 or 4 species- rare 5 species- not recorded (Courtright et al. 2001; Freckman and Virginia 1997; this paper)	bacteria, yeast (Overhoff et al. 1993)	amphimictic (Overhoff et al. 1993)
*Plectus*	*Scottnema*, *Eudorylaimus*, *Geomonhystera*, *Panagrolaimus*	1 species- rare 2 species- most common (usually with *Eudorylaimus*), 3- often 4 species- rare 5 species- not recorded	bacteria (Wharton and Brown 1989)	usually unisexual (parthenogenic), males do exist but are very rare (Andrássy 2008; Kito et al. 1991; Vinciguerra 1994)
*Eudorylaimus*	*Scottnema*, *Plectus*, *Geomonhystera*, *Panagrolaimus*	1 species- not recorded 2 species- most common (usually with *Scottnema* or *Plectus*), 3- often 4 species- rare 5 species- not recorded	*Eudorylaimus* are thought to feed on fungi, unicellular algae and soil invertebrates (Raymond et al. 2013a; Yeates et al. 1993); presence of chloroplasts in esophagus (Wall 2007)	amphimictic (*Eudorylaimus antarcticus*) (Yeates 1970)
*Panagrolaimus*	*Scottnema* (rare, only in Dry Valleys), *Eudorylaimus*, *Plectus*	1 species- common (most common outside of Victoria Land) 2 species- rare 3- common (with *Eudorylaimus* and *Plectus*), 4 species- rare, only in Dry Valleys 5 species- not recorded (Porazinska et al. 2002a; this paper)	bacteria (Wharton 1994; Wharton and Barclay 1993)	amphimictic (Timm 1971)
*Geomonhystera*	*Scottnema*, *Eudorylaimus*, *Plectus*	1 species- not recorded 2 species- often (with *Eudorylaimus antarcticus*) 3- most common (with *Scottnema lindsayae* and *Eudorylaimus antarcticus*) 4 species- often 5 species- not recorded (this paper)	algae, fungi, actinobacteria (Newsham et al. 2004)	amphimictic (Andrássy 1981; Timm 1971)

## Results and discussion

Only five genera of terrestrial nematodes are recorded from Victoria Land Antarctica: *Scottnema*, *Plectus*, *Eudorylaimus*, *Panagrolaimus*, and *Geomonhystera*. For some genera species delimitation remains unresolved (Andrássy 1998; Velasco-Castrillón and Stevens 2014).

### *Scottnema* (Rhabditida: Cephalobidae)

*Scottnema* is an exclusively Antarctic genus comprised of only one species, *Scottnema lindsayae* (Timm 1971). *Scottnema lindsayae* (synonymous with *Scottnema lindsayi*) is thought to have evolved from a common ancestor of the genus *Acrobeles* (Shishida and Ohyama 1986), with a recent phylogenetic analysis placing the genus *Stegelletina* as its closest relative (Boström et al. 2011). *Scottnema lindsayae* is the most southerly known occurring nematode in the world, found as far south as Mt Harcourt (83°08.99'S, 163°21.81'E) near the base of the Beardmore Glacier (Adams et al. 2007).

**Biogeographic distribution.** *Scottnema lindsayae* is the dominant nematode of Victoria Land (Table 1) based on abundance and widespread distribution in numerous samples from the McMurdo Dry Valleys (Courtright et al. 2001; Freckman and Virginia 1990; 1993, 1997; Moorhead et al. 1999; Porazinska et al. 2002b; Powers et al. 1995b; Powers et al. 1998; Treonis et al. 1999, 2000). *Scottnema lindsayae* was first described in Victoria Land in samples from Wright Valley and the southern coastal region (Marble Point, Strand Moraines) (Timm 1971) and has since been recorded in the northern coastal region occurring as far north as Luther Cirque (72°22.20'S, 169°53.10'E) (Table 1).

*Scottnema lindsayae* also occurs on two islands off the coast of Victoria Land: Ross Island (Porazinska et al. 2002a; Sinclair 2001; Timm 1971) and Kay Island (Vinciguerra 1994). On the opposite side of Antarctica, Shishida and Ohyama (1986) report *Scottnema lindsayae* from Rundvågshetta, East Ongul Island (69°01'S, 39°58'E), and Mouratov et al. (2001) report *Scottnema lindsayae* near Machu Picchu station (62°05.51'S, 58°28.21'W) on the coast of Admiralty Bay, although Andrássy (1998) questions this report.

**Habitat.** *Scottnema lindsayae* survives in a wide range of terrestrial habitats (Table 1). In Victoria Land *Scottnema lindsayae* occurs most commonly in dry, bare and sandy or rocky soils and has been found at 30–40 cm soil depth near south shore of Lake Hoare (Powers et al. 1995b). Less frequently, *Scottnema lindsayae* occurs in the moister habitats such as: snow covered soil (subnivian); near streams and in lake sediments (this paper; Treonis et al. 1999; Vinciguerra 1994); and, under mosses (e.g. *Bryum antarcticum*) (Timm 1971; Vinciguerra 1994). *Scottnema lindsayae* has also been found associated with an algal mat (Timm 1971) but whether the algal mat was from soil, a lake or a stream is unknown.

In comparison with other nematodes of Victoria Land, *Scottnema lindsayae* occurs most frequently and at greater abundances in soil habitats with lower moisture, higher pH, higher EC, and higher inorganic C (Courtright et al. 2001; Freckman and Virginia 1997; Moorhead et al. 1999; Porazinska et al. 2002b; Powers et al. 1998; Treonis et al. 1999). In these habitat types, *Scottnema lindsayae* may comprise >99% of invertebrates present (Treonis et al. 1999, 2002), and may be the only invertebrate present. Treonis et al. (2000) found that *Scottnema lindsayae* becomes anhydrobiotic in coarse textured Dry Valley soils at a gravimetric soil moisture threshold of ~2%. In a study of 32 samples from one site on King George Island (62°05.51'S, 58°28.21'W), Mouratov et al. (2001) suggested soil moisture content may be one of the main factors determining the distribution of *Scottnema lindsayae* and found that the species has a preference for soil moisture of 2–5%. Many studies in the McMurdo Dry Valleys (Barrett et al. 2006c; Courtright et al. 2001; Porazinska et al. 2002b; Powers et al. 1998) have identified a relationship between greater abundance of *Scottnema lindsayae* and low soil moisture. *Scottnema lindsayae* tolerates a wide range of soil moistures, but is typically absent from flowing meltstreams and saturated soils. Interactions between soil moisture and salinity are complex and create changing osmotic conditions in soils. In a comparative study of dry soil and moist soil under snowpacks no correlation was found between *Scottnema lindsayae* and soil moisture (Gooseff et al. 2003), which could be attributed to changing osmotic potential and salinity. Soil salinity factors (EC and pH) have a significant influence on the distribution of *Scottnema lindsayae* in the Dry Valleys (Freckman and Virginia 1997; Poage et al. 2008; Porazinska et al. 2002b). For example, *Scottnema lindsayae* are found predominantly in soils with an EC<700 mS cm^-1^ (Courtright et al. 2001; Nkem et al. 2006a; Poage et al. 2008), and appear unable to tolerate salinity over 4100 mS cm^-1^ (Nkem et al. 2006a).

*Scottnema lindsayae* is recorded at a range of elevations, from the McMurdo Dry Valley floors to about 600 and 1300 m above sea level (at Mt. Suess and Battleship Promentory, respectively) in Victoria Land (Moorhead et al. 2003; Porazinska et al. 2002b; Powers et al. 1998; this paper) and 800 m above sea level outside of Victoria Land (Adams et al. 2006). On Ross Island, *Scottnema lindsayae* occurs in soils located away from penguin rookeries and in soils with ornithogenic inputs (Sinclair and Sjursen 2001), but is absent within rookeries (Porazinska et al. 2002a; Sinclair 2001; Yeates et al. 2009). Similar observations are not recorded for Victoria Land. Other studies recording the presence of *Scottnema lindsayae* outside of Victoria Land have found the nematode amongst mosses (e.g. *Saniona uncinata*) and at King George Island, associated with a perennial plant (*Deschampsia antarctica*) (Mouratov et al. 2001; Shishida and Ohyama 1986; Vinciguerra 1994; Wharton and Brown 1989).

### *Plectus* (Plectida: Plectidae)

Several *Plectus* species have been described from Antarctica: *Plectus antarcticus* (de Man 1904), *Plectus parietinus* (Bastian 1865), *Plectus parvus* (Bastian 1865), *Plectus cirratus* (Bastian 1865), *Plectus belgicae* (de Man 1904), *Plectus murrayi* (Yeates 1970), *Plectus acuminatus* (Bastian 1865) and *Plectus frigophilus* (Kirjanova, 1958). Many species are morphologically similar and several taxonomic statements remain unresolved (Andrássy 1998; Boström 2005; Velasco-Castrillón and Stevens 2014).

**Biogeographic distribution.** Four *Plectus* species have been recorded from Victoria Land: *Plectus antarcticus*, *Plectus frigophilus*, *Plectus murrayi* and *Plectus acuminatus*. Specimens of *Plectus antarcticus* previously described from Victoria Land have been reinterpreted as synonymous with *Plectus murrayi* (and *Plectus belgicae* and *Plectus parvus*) (Kito et al. 1991; Timm 1971; Yeates 1979) such that there are only three currently recognized *Plectus* species in Victoria Land. Most studies have described *Plectus* spp. (*murrayi* and *frigophilus*) from the McMurdo Dry Valleys (Gooseff et al. 2003; Porazinska et al. 2002b; Timm 1971; Wall Freckman and Virginia 1998) with only two studies reporting the occurrence of *Plectus* spp. in other areas of Victoria Land. Bargagli et al. (1997) reported *Plectus* spp. from Edmonson Point and Vinciguerra et al. (1994) found *Plectus antarcticus*, *Plectus frigophilus* and *Plectus acuminatus* at Terra Nova Bay.

In the McMurdo Dry Valleys, only *Plectus murrayi* and *Plectus frigophilus* occur, with *Plectus murrayi* the most abundant and widespread (Table 2). *Plectus murrayi* and *Plectus frigophilus* (Kito et al. 1991; Shishida and Ohyama 1986) are endemic to the Antarctic, but not solely to Victoria Land. Close to Victoria Land, *Plectus murrayi* and *Plectus frigophilus* have been recorded frequently from Ross Island (e.g. Cape Royds, Cape Evans, Cape Crozier, McMurdo Station and Rocky Point) (Dougherty et al. 1960; Murray 1910; Porazinska et al. 2002a; Sinclair 2001; Wharton and Brown 1989) and *Plectus frigophilus* has been recorded on Dunlop Island (Timm 1971; USGS 2003). *Plectus antarcticus* occurs primarily in the maritime, and thus most of the recordings of *Plectus antarcticus* on the continent are assumed to be *Plectus murrayi* (Andrássy 1998).

**Habitat.** All *Plectus* spp. of Victoria Land occupy similar habitats. They are present in soils and sediments (Ayres et al. 2007) and are frequently associated with moist environments and areas supporting algae (e.g. *Nostoc commune*) and moss (e.g. *Bryum antarcticum*) (Table 2). This is consistent with the habitats in which *Plectus* spp. are found in other regions of Antarctica (Andrássy 1998; Andrássy and Gibson 2007; Timm 1971; Wharton and Brown 1989; Yeates 1970).

Soil moisture is a critical factor determining the suitability of habitats for *Plectus* spp. Mouratov et al. (2001) studying *Plectus* spp. in the maritime Antarctic found that they had a preference for soil water content of 7-10%. In the McMurdo Dry Valleys, Courtright et al. (2001) similarly observed *Plectus murrayi* was more likely to occur in habitats with higher moisture contents. This moisture requirement may explain other distributional trends in the occurrence of *Plectus*. In the maritime Antarctic, Mouratov et al. (2001) found *Plectus* spp. abundance to be highest in the deepest soil layer they studied and under the moss, *Saniona uncinata*. In these environments soil moisture is likely to be higher at depth in the soil profile and also under mosses than in bare surface soil habitats. Courtright et al. (2001) also noted that *Plectus murrayi* were more frequently found in soils with higher NH_4_-N, NO_3_-N, organic C, and organic C/organic N ratios than other nematode genera (e.g. *Scottnema*). *Plectus* spp. seem to be sensitive to variation in soil salinity and only occur in soils with low EC (<100 mS cm^-1^), which typically are moist environments where salts have been leached from the soil or sediment. Shishida and Ohyama (1986) noted that *Plectus frigophilus* seems to prefer habitats of fresh water algae to those of mosses.

### *Eudorylaimus* (Dorylaimida: Dorylaimidae)

There are six recognized *Eudorylaimus* species endemic to continental Antarctica: *Eudorylaimus antarcticus* (Yeates, 1970), *Eudorylaimus nudicaudatus* (Heyns, 1993), *Eudorylaimus shirasei* (Kito, Shishida & Ohyama, 1996), *Eudorylaimus glacialis* (Andrássy, 1998), *Eudorylaimus quintus* (Andrássy 2008) and *Eudorylaimus sextus* (Andrássy 2008). *Eudorylaimus antarcticus* is nearly universally reported as the sole species recovered from Victoria Land, but it has been suggested that this species is widely codistributed with *Eudorylaimus glacialis* (Andrássy 2008). We report both where two distinct morphotypes were observed.

**Biogeographic distribution.** *Eudorylaimus antarcticus* is widely distributed within Victoria Land (Table 3). Steiner (1916) described the original specimens, which were collected by the Discovery Expedition from Discovery Bay (no notes were made on habitat). Later studies list *Eudorylaimus antarcticus* from locations throughout the McMurdo Dry Valleys, (reported most frequently from Taylor Valley) and in northern Victoria Land at Edmonson Point and Terra Nova Bay (Table 3).

Outside of the Victoria Land region, *Eudorylaimus antarcticus* has been reported from several of the maritime islands (Signy, Alexander, King George, Anvers) (e.g. Maslen 1982; Mouratov et al. 2001; Shishida and Ohyama 1989; Spaull 1973a, b; Wharton and Block 1993). Andrássy (1998, 2008), in contrast, argues for a more restricted distribution within Victoria Land (Andrássy 2008).

**Habitat.** *Eudorylaimus antarcticus* in Victoria Land occurs at varying elevation and most commonly in soils and in lake sediments. The genus has also frequently been associated with algal mats, both dry and moist found in meltwater, streambeds and lakes. *Eudorylaimus antarcticus* has been reported less frequently in areas of moss and from soils. In contrast, outside Victoria Land (e.g. Ross Island) the occurrence of *Eudorylaimus antarcticus* in a moss habitat (e.g. *Bryum argenteum*) is common, but it does not occur in penguin rookeries (on Ross Island or in Victoria Land). In soils of the McMurdo Dry Valleys *Eudorylaimus antarcticus* tends to be found in soils with higher moisture, NH_4_-N, NO_3_-N, organic C, and organic C/organic N ratios, and only occurs in soils with low salinity (EC <100 mS cm^-1^) (Courtright et al. 2001).

### *Panagrolaimus* (Panagrolaimida: Panagrolaimidae)

**Biogeographic distribution.** The Antarctic *Panagrolaimus* consists of two species, *Panagrolaimus magnivulvatus* and *Panagrolaimus davidi* (but see Raymond et al. 2013b). Both are endemic (Andrássy 1998). *Panagrolaimus davidi* is the only species recorded from Victoria Land and its occurrence is rare (see Table 4). Until the present study, the only record of *Panagrolaimus davidi* in Victoria Land was from Marble Point (Timm 1971). The current study shows that *Panagrolaimus davidi* is also present in the northern coastal region of Victoria Land, at Edmonson Point and Cape Hallett and in Miers Valley, one of the McMurdo Dry Valleys. Thus, *Panagrolaimus davidi* occurs most frequently in coastal regions but is not necessarily restricted to them.

*Panagrolaimus davidi* has been recorded from Ross Island (e.g. Freckman and Virginia 1993; Porazinska et al. 2002a; Sinclair 2001; Sinclair and Sjursen 2001; Timm 1971; Wharton and Brown 1989). *Panagrolaimus* spp. have also been reported from several of the maritime islands (summarized in Andrássy 1998 and references therein, see also Raymond et al. 2013b).

**Habitat.** Penguin rookeries and moss-covered soils appear to be the most favorable habitats for *Panagrolaimus davidi* in Victoria Land and are consistent with the habitats where *Panagrolaimus davidi* has been found in other Antarctic ice-free areas (Porazinska et al. 2002a; Sinclair 2001; this paper; Timm 1971; Wharton and Brown 1989). Evidence indicates *Panagrolaimus davidi* occurs in habitats of high primary productivity and soil organic matter (as does *Panagrolaimus magnivulvatus*) regardless of its source of origin (e.g. mosses or penguin guano) though it is primarily associated with penguin rookeries (Porazinska et al. 2002a; Sinclair and Sjursen 2001). The presence of *Panagrolaimus davidi* is strongly correlated with organic carbon, organic nitrogen, chlorophyll *a* (a measure of primary productivity) and ammonium (Porazinska et al. 2002a; Sinclair and Sjursen 2001). The species is also more abundant in the highly productive areas of moss and algae along snow melt streams than in adjacent soils (Sinclair and Sjursen 2001).

### *Geomonhystera* (Monhysterida: Monhysteridae)

Several nematode species originally described as *Monhystera* were redescribed by Andrássy in 1981 as *Geomonhystera*. Among these was *Monhystera villosa* from the Antarctic (Timm 1971), which Andrássy subsequently redescribed as a new species, *Geomonhystera antarcticola* (Andrássy 1998). It is the only known species of *Geomonhystera* on the continent, thus, we report all published observations of the genus from Victoria Land as *Geomonhystera antarcticola*.

**Biogeographic distribution.** *Geomonhystera antarcticola* are generally rare, and along with *Panagrolaimus davidi* are the least abundant and most patchily distributed of all nematodes in Victoria Land. Other species of *Geomonhystera* occur in the islands of the maritime Antarctic (Signy, Coronation, Elephant, Intercurrence and Galindez) where *Geomonhystera antarcticola* is one of the most common nematode species (Maslen 1981; Newsham et al. 2004; Spaull 1973a, b, c). They were originally recorded as Monhysterid genus A. and renamed as *Monhystera villosa* by Maslen (1979). Newsham et al. (2004) identified specimens from Signy Island as *Geomonhystera villosa*.

Sohlenius et al. recorded *Monhystera* from the Nunataks of Dronning Maud Land, East Antarctica (Sohlenius et al. 1995, 1996), and they have also been recovered from Macquarie Island of the Sub-Antarctic (Bunt 1954) and Signy Island of the maritime Antarctic (Caldwell 1981; Maslen 1981; Spaull 1973a, b, c; Wharton and Block 1993) but only identified as *Monhystera* spp., so it is unknown whether these nematodes could also be *Geomonhystera*. Some previously recorded *Monhystera* of the subantarctic (*Monhystera vulgaris*, and *Monhystera filiformis*) (Bunt 1954) are not *Geomonhystera* but more likely *Eumonhystera* (Andrássy 1981) or *Halomonhystera* (Andrássy 2006).

**Habitat.** The habitat of *Geomonhystera* in Victoria Land differs from that of *Geomonhystera* as described by Andrássy (1981), and for *Geomonhystera* of the maritime Antarctic, and *Monhystera* spp. of the maritime Antarctic and Dronning Maud Land. In Victoria Land, *Geomonhystera* are similarly found in soil, but have also been associated with algal mats (e.g. Timm 1971; Wharton and Brown 1989) and moss carpets (Andrássy 1998, this paper). *Monhystera* spp. described from the Nunataks of Dronning Maud Land (Sohlenius et al. 1995; 1996) have only been found under lichens but there is no apparent link between *Geomonhystera* of Victoria Land and lichens.

## Discussion

Nematode diversity in Victoria Land is low compared to the Antarctic Peninsula, but the presence of a few cryptic species is likely (Barrett et al. 2006c; Raymond et al. 2013b). Extensive sampling across broader geographic scales, combined with molecular techniques will likely recover additional species from both locations. With the exception of *Panagrolaimus davidii* and *Geomonhystera* spp., all species are widely distributed throughout Victoria Land, from the south coast and the most southern McMurdo Dry Valleys to the northern coastal region. This distribution suggests that their dispersal is ubiquitous and primarily by wind while in anhydrobiois (Nkem et al. 2006b), and it is the suitability of the soil habitat that determines the likelihood of population and community establishment and functioning (Virginia and Wall 1999).

Our knowledge of nematode biodiversity, distribution, and function in Victoria Land is based on clusters of studies from a few distinct regions, such as the McMurdo Dry Valleys, and far northern coastal Victoria Land, which are accessible from established research stations. The rest of Victoria Land (including other inland ice-free areas) has been largely inaccessible. Studies throughout the McMurdo Dry Valleys are also patchy with some valleys being studied heavily (e.g. Taylor Valley) whilst others (e.g. Barwick Valley) have barely been investigated. More undescribed nematodes may occur in these less studied regions.

## Conclusions

Habitat suitability for each nematode species is determined primarily by variations in soil factors such as quantities and types of organic material, moisture and salinity (Nkem et al. 2006a; Virginia and Wall 1999). *Scottnema lindsayae* is the most abundant and widespread nematode and has a unique tolerance for a wide range of extreme soil habitats, and it is also the most tolerant to low soil moisture and high salinity of all the nematode species studied. These conditions define the most common soil habitats throughout the cold desert ecosystems of Victoria Land and explain the high abundance and broad distribution of *Scottnema lindsayae* throughout the region. There are less extensive suitable habitats available in Victoria Land for *Plectus* spp. and *Eudorylaimus antarcticus* as their distributions are limited to habitats with higher moisture, greater organic material and lower salinity. *Panagrolaimus davidii* has a very limited biogeographic distribution, almost entirely restricted to coastal Victoria Land. This species is found in habitats with high primary productivity, of which there are few. Factors defining suitable habitats and the biogeographic distribution of *Geomonhystera* spp. in Victoria Land are the least understood, largely due to very low abundance and limited occurrence, although they have been recovered from sites across Victoria Land. There appears to be an association with algae but little else is known of their habitat requirements.

We have made considerable progress in understanding the basic relationships between soil properties and the distribution of the key nematode taxa throughout Victoria Land. Suitable habitats can be defined by moisture, salinity, organic matter and nutrient content, and the interactions between these factors. Manipulations of soil moisture and field observations of environmental change during pulse warming events show that nematode community composition can respond on time scales of seasons to decades (Ayres et al. 2010; Doran et al. 2002). The climate of Victoria Land is expected to change with warmer conditions (Adams et al. 2009; Jones et al. 1998; Salby et al. 2011; Solomon et al. 2007; Steig et al. 2009; Thompson and Solomon 2002) leading to increasing soil moisture, redistribution of salts, and potentially higher productivity (Gooseff et al. 2011; Nielsen et al. 2012). These changes may alter the spatial distributions of suitable habitats for individual nematode species and/or alter population size and community diversity (Nielsen et al. 2011b). Studies have shown the important role of nematodes in carbon cycling, suggesting that changes in nematode biogeography will be linked with changes in ecosystem functioning in Antarctic soils (Barrett et al. 2008).

The nematofauna of Victoria Land are capable of long distance dispersal by wind (Nkem et al. 2006b) but the Antarctic continent is effectively isolated from source populations elsewhere in the southern hemisphere (Convey et al. 2008; Convey and Stevens 2007). This leaves anthropogenic dispersal by way of tourists and scientists as the primary mechanism for the movement of alien species to Antarctica (Chown et al. 2012a). From a field sample collected in Wright Valley in the 2011-2012 field season, we recovered an individual living female *Cuticularia fermata*, a nematode heretofore known only from South Orkney Island (subantarctic island). Whether this specimen was transported to the site on clothing or equipment used by scientists or if there are established, low-density, isolated populations in the area is unknown. It is highly likely that the frequency of nematode introductions to Victoria Land will increase as tourism and scientific research increases (Chown et al. 2012a). There is a growing international consensus that action is needed to reduce the potential introductions of invasive soil species to continental Antarctica and the Peninsula and maritime regions (Chown et al. 2012b). A greater knowledge of nematode biogeography will be essential in understanding how to protect special soil habitats to preserve existing biodiversity and to prevent the introduction of non-native species and the potential harm they cause to the unique soil ecosystems of Antarctica.

## References

[B1] Adams B Arthern R Atkinson A Barbante C Bargagli R Bergstrom D Bertler N Bindschadler R Bockheim JG Boutron C Bromwich D Chown S Comiso J Convey P Cook A di Prisco G Fahrback E Fastook J Forcarda J Gili J-M Gugliemin M Gutt J Hellmer H Hennion F Heywood K Hodgson D Holland D Hong S Huiskes A Isla E Jacobs S Jones A Lenton A Marshall G Mayewski P Meredith M Metzl N Monaghan A Naveira-Garabato A Newsham K Orejas C Peck L Portner H-O Rintoul S Robinson S Roscoe H Rossi S Scambos T Shanklin J Smetacek V Speer K Stevens MI Summerhayes C Trathan P Turner J van der Veen K Vaughan D Verde C Webb D Wiencke C Woodworth P Worby T Worland R Yamanouchi T (2009) The Instrumental Period. In: Turner J Bindschadler R Convey P di Prisco G Fahrbach E Gutt J Hodgson D Mayewski P Summerhayes C (Eds) Antarctic Climate Change and the Environment. Scientific Committee on Antarctic Research, Cambridge, UK, 183–298

[B2] Adams BJ Bardgett RD Ayres E Wall DH Aislabie J Bamforth S Bargagli R Cary C Cavacini P Connell L Convey P Fell JW Frati F Hogg ID Newsham KK O’Donnell A Russell N Seppelt RD Stevens MI (2006) Diversity and distribution of Victoria Land biota. Soil Biology and Biochemistry 38: 3003–3018. doi: 10.1016/j.soilbio.2006.04.030

[B3] Adams BJ Wall DH Gozel U Dillman AR Chaston JM Hogg ID (2007) The southernmost worm, *Scottnema lindsayae* (Nematoda): diversity, dispersal and ecological stability. Polar Biology 30: 809–815. doi: 10.1007/s00300-006-0241-3

[B4] Adhikari B Wall D Adams B (2009) Desiccation survival in an Antarctic nematode: Molecular analysis using expressed sequenced tags. BMC Genomics 10: . doi: 10.1186/1471-2164-10-69 10.1186/1471-2164-10-69 PMC2667540 19203352

[B5] Adhikari BN Adams BJ (2011) Molecular analysis of desiccation survival in Antarctic nematodes. In: Perry RN Wharton DA (Eds) Molecular and Physiological Basis of Nematode Survival. CABI International, Wallingford, 205–232. doi: 10.1079/9781845936877.0205

[B6] Adhikari BN Wall DH Adams BJ (2010) Effect of slow desiccation and freezing on gene transcription and stress survival of an Antarctic nematode. Journal Of Experimental Biology 213: 1803–1812. doi: 10.1242/Jeb.032268 20472766 10.1242/jeb.032268

[B7] Andrássy I (1981) Revision of the order Monhysterida (Nematoda) inhabiting soil and inland waters. Opuscula Zoologica Budapest 17–18: 13–47

[B8] Andrássy I (1998) Nematodes in the sixth continent. Journal of Nematode Morphology and Systematics 1: 107–186

[B9] Andrássy I (2006) *Halomonhystera*, a new genus distinct from *Geomonhystera* Andrássy, 1981 (Nematoda: Monhysteridae). Meiofauna Marina 15: 11–24

[B10] Andrássy I (2008) *Eudorylaimus* species (Nematoda: Dorylaimida) of continental Antarctica. Journal of Nematode Morphology and Systematics 11: 49–66

[B11] Andrássy I Gibson JAE (2007) Nematodes from saline and freshwater lakes of the Vestfold Hills, East Antarctica, including the description of *Hypodontolaimus antarcticus* sp. n. Polar Biology 30: 669–678. doi: 10.1007/s00300-006-0224-4

[B12] Ayres E Nkem JN Wall DH Adams BJ Barrett JE Simmons BL Virginia RA Fountain AG (2010) Experimentally increased snow accumulation alters soil moisture and animal community structure in a polar desert. Polar Biology 33: 897–907. doi: 10.1007/s00300-010-0766-3

[B13] Ayres E Wall DH Adams BJ Barrett JE Virginia RA (2007) Unique Similarity of Faunal Communities across Aquatic–Terrestrial Interfaces in a Polar Desert Ecosystem. Ecosystems 10: 523–535. doi: 10.1007/s10021-007-9035-x

[B14] Ball BA Virginia RA Barrett JE Parsons AN Wall DH (2009) Interactions between physical and biotic factors influence CO2 flux in Antarctic dry valley soils. Soil Biology & Biochemistry 41: 1510–1517. doi: 10.1016/j.soilbio.2009.04.011

[B15] Bamforth SS Wall DH Virginia RA (2005) Distribution and diversity of soil protozoa in the McMurdo Dry Valleys of Antarctica. Polar Biology 28: 756–762. doi: 10.1007/s00300-005-0006-4

[B16] Bargagli R Wynn-Williams D Bersan F Cavacini P Ertz S Frati F Freckman DW Smith Rl Russell N Smith A (1997) Field report, Biotex 1: first BIOTAS expedition (Edmonson Point—Baia Terra Nova, Dec. 10, 1995–Feb. 6, 1996). Newsletter of the Italian Biological Research in Antarctica 1: 42–58

[B17] Barrett JE Virginia RA Hopkins DW Aislabie J Bargagli R Bockheim JG Campbell IB Lyons WB Moorhead DL Nkem JN Sletten RS Steltzer H Wall DH Wallenstein MD (2006a) Terrestrial ecosystem processes of Victoria Land, Antarctica. Soil Biology and Biochemistry 38: 3019–3034. doi: 10.1016/j.soilbio.2006.04.041

[B18] Barrett JE Virginia RA Parsons AN Wall DH (2006b) Soil carbon turnover in the McMurdo Dry Valleys, Antarctica. Soil Biology and Biochemistry 38: 3065–3082. doi: 10.1016/j.soilbio.2006.03.025

[B19] Barrett JE Virginia RA Wall DH Adams BJ (2008) Decline in a dominant invertebrate species contributes to altered carbon cycling in a low-diversity soil ecosystem. Global Change Biology 14: 1734–1744. doi: 10.1111/j.1365-2486.2008.01611.x

[B20] Barrett JE Virginia RA Wall DH Cary SC Adams BJ Hacker AL Aislabie JM (2006c) Co-variation in soil biodiversity and biogeochemistry in northern and southern Victoria Land, Antarctica. Antarctic Science 18: 535–548

[B21] Bastian HC (1865) Monograph on the Anguillulidae, or free nematoids, marine, land, and freshwater; with descriptions of 100 new species. Transactions of the Linnean Society of London 25: 73–184. doi: 10.1111/j.1096-3642.1865.tb00179.x

[B22] Beyer L Bockheim JG Campbell IB Claridge GGC (1999) Genesis, properties and sensitivity of Antarctic Gelisols. Antarctic Science 11: 387–398. doi: 10.1017/S0954102099000498

[B23] Block W Christensen B (1985) Terrestrial Enchytraeidae from South Georgia and the Maritime Antarctic. British Antarctic Survey Bulletin 69: 65–70

[B24] Bloemers GF Hodda M Lambshead PJD Lawton JH Wanless FR (1997) The effects of forest disturbance on diversity of tropical soil nematodes. Oecologia 111: 575–582. doi: 10.1007/s004420050274 10.1007/s004420050274 28308121

[B25] Boag B Yeates GW (1998) Soil nematode biodiversity in terrestrial ecosysytems. Biodiversity and Conservation 7: 617–630. doi: 10.1023/A:1008852301349

[B26] Bockheim JG (1997) Properties and classification of cold desert soils from Antarctica. Soil Science Society of America Journal 61: 224–231. doi: 10.2136/sssaj1997.03615995006100010031x

[B27] Bölter M Beyer L Stonehouse B (2002) Antarctic coastal landscapes: Characteristics, ecology and research. In: Beyer L Bölter M (Eds) Geoecology of Antarctic Ice-Free Coastal Landscapes. Springer-Verlag Berlin Heidelberg, 5–15

[B28] Boström S (2005) Nematodes from Sirmacher Oasis, Dronning, Maud Land, East Antarctica. Russian Journal of Nematology 13: 43–54

[B29] Boström S Holovachov O Nadler SA (2011) Description of *Scottnema lindsayae* Timm, 1971 (Rhabditida: Cephalobidae) from Taylor Valley, Antarctica and its phylogenetic relationship. Polar Biology 34: 1–12. doi: 10.1007/s00300-010-0850-8

[B30] Bunt JS (1954) The soil-inhabiting nematodes of Macquarie Island. Australian Journal of Zoology 2: 264–274. doi: 10.1071/ZO9540264

[B31] Burkins MB Virginia RA Chamberlain CP Wall DH (2000) Origin and distribution of soil organic matter in Taylor Valley, Antarctica. Ecology 81: 2377–2391. doi: 10.1890/0012-9658(2000)081[2377:OADOSO]2.0.CO;2

[B32] Bütschli O (1873) Beiträge zur Kenntnis der freilebenden Nematoden. Nova Acta Ksl Leop Carol Deutsch Akad Naturf 36: 1–144

[B33] Caldwell JR (1981) Biomass and respiration of nematode populations in two moss communities at Signy Island, Maritime Antarctic. OIKOS 37: 160–166. doi: 10.2307/3544460

[B34] Cameron RE King J David CN (1970) Microbiology, ecology and microclimatology of soil sites in Dry Valleys of Southern Victoria Land, Antarctica. In: Holgate MW (Ed) Antarctic Ecology. Academic Press, London, 702–716

[B35] Campbell IB Claridge GC Campbell DI Balks MR (1998) The soil environment of the McMurdo Dry Valleys, Antarctica. In: Priscu JC (Ed) Ecosystem dynamics in a polar desert: the McMurdo Dry Valleys, Antarctica. American Geophysical Union, Washington (DC), 297–322

[B36] Campbell IB Claridge GGC (1966) A sequence of soils from a penguin rookery, Inexpressible Island, Antarctica. New Zealand Journal of Science 9: 361–372

[B37] Chown SL Convey P (2007) Spatial and temporal variability across life’s hierarchies in the terrestrial Antarctic. Philosophical Transactions of the Royal Society of London B 362: 2307–2331. doi: 10.1098/rstb.2006.1949 10.1098/rstb.2006.1949 PMC2443176 17553768

[B38] Chown SL Huiskes AHL Gremmen NJM Lee JE Terauds A Crosbie K Frenot Y Hughes KA Imura S Kiefer K Lebouvier M Raymond B Tsujimoto M Ware C Van de Vijver B Bergstrom DM (2012a) Continent-wide risk assessment for the establishment of nonindigenous species in Antarctica. Proceedings of the National Academy of Sciences of the United States of America 109: 4938–4943. doi: 10.1073/pnas.1119787109 22393003 10.1073/pnas.1119787109 PMC3323995

[B39] Chown SL Lee JE Hughes KA Barnes J Barrett PJ Bergstrom DM Convey P Cowan DA Crosbie K Dyer G Frenot Y Grant SM Herr D Kennicutt MC Lamers M Murray A Possingham HP Reid K Riddle MJ Ryan PG Sanson L Shaw JD Sparrow MD Summerhayes C Terauds A Wall DH (2012b) Challenges to the Future Conservation of the Antarctic. Science 337: 158–159. doi: 10.1126/science.1222821 22798586 10.1126/science.1222821

[B40] Chown SL Van Drimmelen M (1992) Water balance and osmo-regulation in weevil larvae (Coleoptera: Curculionidae: Brachycerinae) from three different habitats on subantarctic Marion Island. Polar Biology 12: 527–532. doi: 10.1007/BF00238192

[B41] Convey P (1996) The influence of environmental characteristics on life history attributes of Antarctic terrestrial biota. Biological Reviews 71: 191–225. doi: 10.1111/j.1469-185X.1996.tb00747.x

[B42] Convey P Gibson JAE Hillenbrand CD Hodgson DA Pugh PJA Smellie JL Stevens MI (2008) Antarctic terrestrial life - challenging the history of the frozen continent? Biological Reviews 83: 103–117. doi: 10.1111/j.1469-185X.2008.00034.x 18429764 10.1111/j.1469-185X.2008.00034.x

[B43] Convey P Stevens MI (2007) Antarctic biodiversity. Science 317: 1877–1878. doi: 10.1126/science.1147261 17901323 10.1126/science.1147261

[B44] Courtright EM Freckman DW Virginia RA Thomas WK (1996) McMurdo LTER: Genetic diversity of soil nematodes in the McMurdo Dry Valleys of Antarctica. Antarctic Journal of the United States 31: 203–204

[B45] Courtright EM Wall DH Virginia RA (2001) Determining habitat suitability for soil invertebrates in an extreme environment: the McMurdo Dry Valleys, Antarctica. Antarctic Science 13: 9–17. doi: 10.1017/S0954102001000037

[B46] Courtright EM Wall DH Virginia RA Frisse LM Vida JT Thomas WK (2000) Nuclear and mitochondrial DNA sequence diversity in the Antarctic nematode *Scottnema lindsayae*. Journal of Nematology 32: 143–153 19270960 PMC2620446

[B47] Crowe JH Hoekstra FA Crowe LM (1992) Anhydrobiosis. Annual Review of Physiology 54: 579–599. doi: 10.1146/annurev.ph.54.030192.003051 10.1146/annurev.ph.54.030192.003051 1562184

[B48] de Man JG (1904) Nématodes libres. Expédition Antarctique Belge. Résultats du voyage du S.Y. Belgica, 1897–1899. Rapports scientifiques Zoologie 8: 1–51

[B49] de Tomasel CM Adams BJ Tomasel FG Wall DH (2013) The life cycle of the Antarctic nematode *Plectus murrayi* under laboratory conditions. Journal of Nematology 45: 39–42 23589658 PMC3625130

[B50] Doran PT Priscu JC Lyons WB Walsh JE Fountain AG McKnight DM Moorhead DL Virginia RA Wall DH Clow GD Fritsen CH McKay CP Parsons AN (2002) Antarctic climate cooling and terrestrial ecosystem response. Letters to Nature 415: 517–520. doi: 10.1038/nature710 10.1038/nature710 11793010

[B51] Dougherty EC Chitwood BG Maggenti AR (1960) Observations on Antarctic freshwater micrometazoa. Anatomical Record 3: 1–350

[B52] Fountain AG Lyons WB Burkins MB Dana GL Doran PT Lewis KJ McKnight DM Moorhead DL Parsons AN Priscu JC Wall DH Wharton RA Virginia RA (1999) Physical controls on the Taylor Valley ecosystem, Antarctica. Bioscience 49: 961–971. doi: 10.2307/1313730

[B53] Frati F Fanciulli PP Carapelli A Dallai R (1997) The Collembola of northern Victoria Land(Antarctica): Distribution and ecological remarks. Pedobiologia 41: 50–55

[B54] Freckman DW Virginia RA (1990) Nematode ecology of the McMurdo Dry Valley ecosystems. Antarctic Journal of the United States 25: 229–230

[B55] Freckman DW Virginia RA (1993) Extraction of nematodes from Dry Valley Antarctic soils. Polar Biology 13: 483–487. doi: 10.1007/BF00233139

[B56] Freckman DW Virginia RA (1997) Low diversity antarctic soil nematode communities: Distribution and response to disturbance. Ecology 78: 363–369. doi: 10.1890/0012-9658(1997)078[0363:LDASNC]2.0.CO;2

[B57] Gooseff MN Barrett JE Doran PT Fountain AG Lyons WB Parsons AN Porazinska DL Virginia RA Wall DH (2003) Snow-patch influence on soil biogeochemical processes and invertebrate distribution in the McMurdo Dry Valleys, Antarctica. Arctic Antarctic and Alpine Research 35: 91–99. doi: 10.1657/1523-0430(2003)035[0091:SPIOSB]2.0.CO;2

[B58] Gooseff MN McKnight DM Doran P Fountain AG Lyons WB (2011) Hydrological Connectivity of the Landscape of the McMurdo Dry Valleys, Antarctica. Geography Compass 5: 666–681. doi: 10.1111/j.1749-8198.2011.00445.x

[B59] Heine JC Speir TW (1989) Ornithogenic soils of the Cape Bird Adelie penguin rookeries, Antarctica. Polar Biology 10: 89–99. doi: 10.1007/BF00239153

[B60] Heyns J (1993) *Eudorylaimus nudicaudatus* sp. n. from Antarctica (Nematoda: Dorylaimoidea). South African Journal of Antarctic Research 23: 33–36

[B61] Horowitz NH Cameron RE Hubbard JS (1972) Microbiology of the Dry Valleys of Antarctica. Science 176: 242–245. doi: 10.1126/science.176.4032.242 17791905 10.1126/science.176.4032.242

[B62] Jones TH Thompson LJ Lawton JH Bezemer TM Bardgett RD Blackburn TM Bruce KD Cannon PF Hall GS Hartley SE Howson G Jones CG Kampichler C Kandler E Ritchie DA (1998) Impacts of rising atmospheric carbon dioxide on model terrestrial ecosystems. Science 280: 441–442. doi: 10.1126/science.280.5362.441 9545223 10.1126/science.280.5362.441

[B63] Kappen L (1993) Lichens in the Antarctic region. In: Friedmann EI (Ed) Antarctic Microbiology. Wiley-Liss, New York, 433–490

[B64] Kirjanova ES (1958) Antarkticheskie predstaviteli presnovodnykh nematod roda *Plectus* Bastian (Nematodes Plectidae). Information Bulletin of the Soviet Antarctic Expedition 3: 101–103

[B65] Kito K Shishida Y Ohyama Y (1991) *Plectus antarcticus* de Man, 1904 and *P. frigophilus* Kirjanova, 1958 (Nematoda: Plectidae), with emphasis on the male, from the Soya Coast, East Antarctica. Nematologica 37: 252–262. doi: 10.1163/187529291X00259

[B66] Kito K Shishida Y Ohyama Y (1996) A new species of the genus *Eudorylaimus* Andrássy, 1959 (Nematoda: Qudsianematidae) from East Antarctica. Polar Biology 16: 163–169. doi: 10.1007/BF02329204

[B67] Lyons WB Fountain A Doran P Priscu JC Neumann K Welch KA (2000) Importance of landscape position and legacy: the evolution of the lakes in Taylor Valley, Antarctica. Freshwater Biology 43: 355–367. doi: 10.1046/j.1365-2427.2000.00513.x

[B68] Maslen NR (1979) Additions to the nematode fauna of the Antarctic region with keys to taxa. British Antarctic Survey Bulletin 49: 207–229

[B69] Maslen NR (1981) The Signy Island terrestrial reference site: XII. Population ecology of nematodes with additions to the fauna. British Antarctic Survey Bulletin 53: 57–75

[B70] Maslen NR (1982) An unidentified nematode-trapping fungus from a pond on Alexander Island. British Antarctic Survey Bulletin 51

[B71] Moorhead DL Barrett JE Virginia RA Wall DH Porazinska D (2003) Organic matter and soil biota of upland wetlands in Taylor Valley, Antarctica. Polar Biology 26: 567–576. doi: 10.2307/1313734

[B72] Moorhead DL Doran PT Fountain AG Lyons WB McKnight DM Priscu JC Virginia RA Wall DH (1999) Ecological legacies: Impacts on ecosystems of the McMurdo Dry Valleys. Bioscience 49: 1009–1019

[B73] Mouratov S Lahav I Barness G Steinberger Y (2001) Preliminary study of the soil nematode community at Machu Picchu Station, King George Island, Antarctica. Polar Biology 24: 545–548. doi: 10.1007/s003000100242

[B74] Murray J (1910) Microscopic life at Cape Royds. British Antarctic Expedition, 1907–1909 Reports on the Scientific Investigations 1: 17–22

[B75] Newsham KK Rolf J Pearce DA Strachan RJ (2004) Differing preferences of Antarctic soil nematodes for microbial prey. European Journal of Soil Biology 40: 1–8. doi: 10.1016/j.ejsobi.2004.01.004

[B76] Nielsen UN Wall DH Adams BJ Virginia RA (2011a) Antarctic nematode communities: observed and predicted responses to climate change. Polar Biology: 1701–1711. doi: 10.1007/s00300-011-1021-2

[B77] Nielsen UN Wall DH Adams BJ Virginia RA Ball BA Gooseff MN McKnight DM (2012) The ecology of pulse events: insights from an extreme climatic event in a polar desert ecosystem. Ecosphere 3: art17. doi: 10.1890/ES11-00325.1

[B78] Nielsen UN Wall DH Li G Toro M Adams BJ Virginia RA (2011b) Nematode communities of Byers Peninsula, Livingston Island, maritime Antarctica. Antarctic Science 23: 349–357. doi: 10.1017/s0954102011000174

[B79] Nkem JN Virginia RA Barrett JE Wall DH Li G (2006a) Salt tolerance and survival thresholds for two species of Antarctic soil nematodes. Polar Biology 29: 643–651. doi: 10.1007/s00300-005-0101-6

[B80] Nkem JN Wall DH Virginia RA Barrett JE Broos EJ Porazinska DL Adams BJ (2006b) Wind dispersal of soil invertebrates in the McMurdo Dry Valleys, Antarctica. Polar Biology 29: 346–352. doi: 10.1007/s00300-005-0061-x

[B81] Overhoff A Freckman DW Virginia RA (1993) Life cycle of the microbivorous Antarctic Dry Valley nematode *Scottnema lindsayae* (Timm 1971). Polar Biology 13: 151–156. doi: 10.1007/BF00238924

[B82] Parsons AN Barrett JE Wall DH Virginia RA (2004) Soil carbon dioxide flux in Antarctic Dry Valley ecosystems. Ecosystems 7: 286–295. doi: 10.1007/s10021-003-0132-1

[B83] Peat HJ Clarke A Convey P (2007) Diversity and biogeography of the Antarctic flora. Journal of Biogeography 34: 132–146. doi: 10.1111/j.1365-2699.2006.01565.x

[B84] Poage MA Barrett JE Virginia RA Wall DH (2008) The influence of soil geochemistry on nematode distribution, McMurdo Dry Valleys, Antarctica. Arctic, Antarctic, and Alpine Research 40: 119–128. doi: 10.1657/1523-0430(06-051)[POAGE]2.0.CO;2

[B85] Porazinska DL Wall DH Virginia RA (2002a) Invertebrates in ornithogenic soils on Ross Island, Antarctica. Polar Biology 25: 569–574. doi: 10.1007/s00300-002-0386-7

[B86] Porazinska DL Wall DH Virginia RA (2002b) Population age structure of nematodes in the Antarctic Dry Valleys: Perspectives on time, space, and habitat suitability. Arctic, Antarctic, and Alpine Research 34: 159–168. doi: 10.2307/1552467

[B87] Powers LE Freckman DW Ho M Virginia RA (1995a) McMurdo LTER: Soil properties associated with nematode distribution along an elevational transect in Taylor Valley, Antarctica. Antarctic Journal - Review 30: 282–287

[B88] Powers LE Freckman DW Virginia RA (1994a) Depth distribution of soil nematodes in Taylor Valley, Antarctica. Antarctic Journal of the United States 29: 175–176

[B89] Powers LE Freckman DW Virginia RA (1995b) Spatial distribution of nematodes in polar desert soils of Antarctica. Polar Biology 15: 325–333. doi: 10.1007/BF00238482

[B90] Powers LE Ho M Freckman DW Virginia RA (1994b) McMurdo LTER: Soil and nematode distribution along elevational gradient in Taylor valley, Antarctica. Antarctic Journal of the United States 29: 228–229

[B91] Powers LE Ho MC Freckman DW Virginia RA (1998) Distribution, community structure, and microhabitats of soil invertebrates along an elevational gradient in Taylor Valley, Antarctica. Arctic and Alpine Research 30: 133–141. doi: 10.1007/BF00238482

[B92] Raymond MR Wharton DA Marshall CJ (2013a) Factors determining nematode distributions at Cape Hallett and Gondwana station, Antarctica. Antarctic Science 25: 347–357. doi: 10.1017/s0954102012001162

[B93] Raymond MR Wharton DA Marshall CJ (2013b) Nematodes from the Victoria Land coast, Antarctica and comparisons with cultured *Panagrolaimus davidi*. Antarctic Science: 1–8. doi: 10.1017/S0954102013000230

[B94] Salby M Titova E Deschamps L (2011) Rebound of Antarctic ozone. Geophysical Research Letters 38: . doi: 10.1029/2011GL047266

[B95] Shishida Y Ohyama Y (1986) A note on the terrestrial nematodes around Syowa Station, Antarctica. Memoirs of the National Institute of Polar Research 44: 259–260

[B96] Shishida Y Ohyama Y (1989) A note on the terrestrial nematodes around Palmer Station, Antarctica. Proceedings of the NIPR Symposium on Polar Biology 2: 223–224

[B97] Simmons BL Wall DH Adams BJ Ayres E Barrett JE Virginia RA (2009) Long-term experimental warming reduces soil nematode populations in the McMurdo Dry Valleys, Antarctica. Soil Biology and Biochemistry 41: 2052–2060. doi: 10.1016/j.soilbio.2009.07.009

[B98] Sinclair BJ (2001) On the distribution of terrestrial invertebrates at Cape Bird, Ross Island, Antarctica. Polar Biology 24: 394–400. doi: 10.1007/s003000000223

[B99] Sinclair BJ Sjursen H (2001) Terrestrial invertebrate abundance across a habitat transect in Keble Valley, Ross Island, Antarctica. Pedobiologia 45: 134–145. doi: 10.1078/0031-4056-00075

[B100] Sohlenius B Boström S Hirschfelder A (1995) Nematodes, rotifers and tardigrades from nunataks in Dronning Maud Land, East Antarctica. Polar Biology 15: 51–56. doi: 10.1007/BF00236124

[B101] Sohlenius B Boström S Hirschfelder A (1996) Distribution patterns of microfauna (nematodes, rotifers and tardigrades) on nunataks in Dronning Maud Land, East Antarctica. Polar Biology 16: 191–200. doi: 10.1007/bf02329207

[B102] Solomon S, Intergovernmental Panel on Climate Change, Intergovernmental Panel on Climate Change Working Group I. (2007) Climate Change 2007: the physical science basis: contribution of Working Group I to the Fourth Assessment Report of the Intergovernmental Panel on Climate Change. Cambridge University Press, Cambridge, New York, 996 pp

[B103] Spaull VW (1973a) Distribution of soil nematodes in the maritime Antarctic. British Antarctic Survey Bulletin 37: 1–6

[B104] Spaull VW (1973b) Qualitative and quantitative distribution of soil nematodes of Signy Island, South Orkney Islands. British Antarctic Survey Bulletin 33–34: 177–184

[B105] Spaull VW (1973c) Seasonal variation in numbers of soil nematodes at Signy Island, South Orkney Islands. British Antarctic Survey Bulletin 33–34: 47–56

[B106] Steig EJ Schneider DP Rutherford SD Mann ME Comiso JC Shindell DT (2009) Warming of the Antarctic ice-sheet surface since the 1957 International Geophysical Year. Nature 457: 459–463. doi: 10.1038/nature07669 19158794 10.1038/nature07669

[B107] Steiner G (1916) Beiträge zur geographischen Verbreitung freilebender Nematoden. 46: 311–335

[B108] Stevens MI Hogg ID (2002) Long-term isolation and recent range expansion from glacial refugia revealed for the endemic springtail *Gomphiocephalus hodgsoni* from Victoria Land, Antarctica. Molecular Ecology 12: 2357–2369. doi: 10.1046/j.1365-294X.2003.01907.x 12919474 10.1046/j.1365-294x.2003.01907.x

[B109] Thompson DWJ Solomon S (2002) Interpretation of recent Southern Hemisphere climate change. Science 296: 895–899. doi: 10.1126/science.1069270 11988571 10.1126/science.1069270

[B110] Timm RW (1971) Antarctic soil and freshwater nematodes from the McMurdo Sound region. Proceedings of the Helminthological Society of Washington 38: 42–52

[B111] Treonis AM Wall DH Virginia RA (1999) Invertebrate biodiversity in Antarctic Dry Valley soils and sediments. Ecosystems 2: 482–492. doi: 10.1007/s100219900096

[B112] Treonis AM Wall DH Virginia RA (2000) The use of anhydrobiosis by soil nematodes in the Antarctic Dry Valleys. Functional Ecology 14: 460–467. doi: 10.1046/j.1365-2435.2000.00442.x

[B113] Treonis AM Wall DH Virginia RA (2002) Field and microcosm studies of decomposition and soil biota in a cold desert soil. Ecosystems 5: 159–170. doi: 10.1007/s10021-001-0062-8

[B114] USGS Atlas of Antarctic Research.

[B115] Velasco-Castrillón A Stevens MI (2014) Morphological and molecular diversity at a regional scale: A step closer to understanding Antarctic nematode biogeography. Soil Biology & Biochemistry 70: 272–284. doi: 10.1016/j.soilbio.2013.12.016

[B116] Vinciguerra MT (1994) *Metacrolobus festonatus* gen. n. sp. n. and. *Scottnema lindsayae* Timm, 1971 (Nemata: Cephalobidae) from Subantarctic and Antarctic regions with proposal of the new subfamily Metacrolobinae. Fundamental and Applied Nematology 17: 175–180

[B117] Vinciguerra MT Binda MG Pilato G (1994) Nematodes and tardigrades of Antarctica: Results of the researches conducted in 1988–1991. In: Battaglia B Bisol PM Varotto V (Eds) Proceedings of the 2nd Meeting on Antarctic Biology. Padova, Dipartimento di Biologia dell’Università, 26–28 February 1992, 83–88

[B118] Virginia RA Wall DH (1999) How soils structure communities in the Antarctic Dry Valleys. Bioscience 49: 973–983. doi: 10.2307/1313731

[B119] Wall DH (2004) Sustaining biodiversity and ecosystem services in soils and sediments. Island Press, Washington, DC, 275 pp

[B120] Wall DH (2005) Biodiversity and ecosystem functioning in terrestrial habitats of Antarctica. Antarctic Science 17: 523–531. doi: 10.1017/s0954102005002944

[B121] Wall DH (2007) Global change tipping points: above- and below-ground biotic interactions in a low diversity ecosystem. Philosophical Transactions of the Royal Society of London B 362: 2291–2306. doi: 10.1098/rstb.2006.1950 10.1098/rstb.2006.1950 PMC2443177 17553769

[B122] Wall Freckman DW Virginia RA (1998) Soil biodiversity and community structure in the McMurdo Dry Valleys, Antarctica. In: Priscu JC (Ed) Ecosystem Dynamics in a Polar Desert: The McMurdo Dry Valleys, Antarctica. American Geophysical Union, Washington, DC, 323–336

[B123] Wharton DA (1994) Freezing avoidance in the eggs of the Antarctic nematode *Panagrolaimus davidi*. Fundamental and Applied Nematology 17: 239–243

[B124] Wharton DA (2003) The environmental physiology of Antarctic terrestrial nematodes: a review. Journal of Comparative Physiology B-Biochemical Systemic and Environmental Physiology 173: 621–628. doi: 10.1007/s00360-003-0378-0 10.1007/s00360-003-0378-0 14615899

[B125] Wharton DA (2010) Osmoregulation in the Antarctic nematode *Panagrolaimus davidi*. Journal Of Experimental Biology 213: 2025–2030. doi: 10.1242/jeb.041202 20511515 10.1242/jeb.041202

[B126] Wharton DA Barclay S (1993) Anhydrobiosis in the free-living Antarctic nematode *Panagrolaimus davidi* (Nematoda: Rhabditida). Fundamental and Applied Nematology 16: 17–22

[B127] Wharton DA Block W (1993) Freezing tolerance in some Antarctic nematodes. Functional Ecology 7: 578–584. doi: 10.2307/2390134

[B128] Wharton DA Brown IM (1989) A survey of terrestrial nematodes from the McMurdo Sound region, Antarctica. New Zealand Journal of Zoology 16: 467–470. doi: 10.1080/03014223.1989.10422914

[B129] Yeates GW (1970) Two Terrestrial Nematodes from the McMurdo Sound Region Antarctica, with a Note on *Anaplectus arenicola* Killick, 1964. Journal of Helminthology 44: 27–34. doi: 10.1017/S0022149X00021416

[B130] Yeates GW (1979) Terrestrial nematodes from the Bunger Hills and Gaussberg, Antarctica. New Zealand Journal of Zoology 6: 641–643. doi: 10.1080/03014223.1979.10428408

[B131] Yeates GW Bongers T De Goede RGM Freckman DW Georgieva SS (1993) Feeding habits in nematode families and genera - an outline for soil ecologists. Journal of Nematology 25: 315–331 19279775 PMC2619405

[B132] Yeates GW Scott MB Chown SL Sinclair BJ (2009) Changes in soil nematode populations indicate an annual life cycle at Cape Hallett, Antarctica. Pedobiologia 52: 375–386. doi: 10.1016/j.pedobi.2009.01.001

